# Redefining the Health Risk of Battery Materials Through a Biologically Transformed Metal Mixture

**DOI:** 10.1002/advs.202523469

**Published:** 2026-01-30

**Authors:** Ze Zhang, Gan Miao, Xueyu Zhang, Zhao Shu, Dawei Lu, Yujie Song, Shanfa Yu, Qian Liu, Yang Song, Rong Zhang, Xiaoting Jin, Yuxin Zheng

**Affiliations:** ^1^ Department of Occupational Health and Environmental Health School of Public Health Qingdao University Qingdao China; ^2^ State Key Laboratory of Environmental Chemistry and Ecotoxicology Research Center for Eco‐Environmental Sciences Chinese Academy of Sciences Beijing China; ^3^ Department of Public Health Henan Medical College Zhengzhou China; ^4^ Department of Toxicology School of Public Health Hebei Medical University Shijiazhuang China

**Keywords:** biological transformation, health risk paradigm, lithium‐ion battery, metal mixture toxicology, sustainable energy materials

## Abstract

The global transition to electric vehicles hinges on lithium‐ion batteries, yet the health risks of their core components, such as nickel‐manganese‐cobalt (NCM) cathodes, remain a critical and misunderstood gap, threatening a truly sustainable energy transition. Herein, we reveal that inhaled NCM particles undergo sustained lysosomal dissolution, transforming into metal mixtures whose composition mirrors the parent material. Crucially, we decipher the unique toxicological interactions within this biologically generated mixture—antagonism from Ni/Co and synergy from Mn. This fundamental discovery of NCM's biological fate unlocks accurate risk assessment. Building on this mechanistic insight, we identified the Integrated Addition and Interaction (IAI) model as the framework capable of capturing complex interactions. Applying this model to real‐world exposure data uncovers moderate yet significant population‐level health risks. Our work establishes a transformative, evidence‐based paradigm that connects in‐body material transformation to real‐world health outcomes, providing the scientific foundation to ensure that the clean energy transition is not only green but fundamentally safe for human well‐being.

## Graphical abstract

Inhaled NCM particles undergo lysosomal degradation, releasing complex ion mixtures that induce systemic impact. The impact is determined by a critical balance between antagonistic Ni‐Co interactions and synergistic Mn effects. To capture these complexities in risk assessment, we develop an IAI model, ensuring a more accurate quantitative risk assessment. Application to NCM high‐exposure populations indicates major moderate health risks.

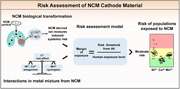

## 1 Introduction

The global transition to electric vehicles, a cornerstone of a sustainable future, is driven by a fundamental paradox: its core technology, the lithium‐ion battery, may harbor an unrecognized threat to human health [1, 2]. At the heart of this revolution lies the nickel‐manganese‐cobalt (NCM) cathode, a heterometallic material that now dominates over 64 % of the electric vehicle market [3, 4]. However, as production and application of NCM escalate, a critical blind spot in our pursuit of a green transition has emerged: the widespread release of NCM particles into the environment and workplaces, creating a new class of emerging pollutants [5]. Inhalation of these particles, which can lead to elevated systemic levels of Li, Ni, Co, and Mn in exposed individuals, represents a primary exposure route [6, 7]. Yet, the fundamental question of their true health impact remains unanswered [8, 9], posing a direct threat to the promise of a truly sustainable energy transition.

While existing studies have documented the adverse health effects associated with exposure to individual metal components of NCM [10], our understanding of the risk posed by the intact material is critically incomplete. The core of the problem lies in a fundamental unknown: what is the biological fate of NCM once inside the human body? Analogous to its behavior in the environment, the material's toxicity is likely governed not by the particle itself, but by its in‐body transformation and the release of its constituent metals [11, 12]. This knowledge gap is not merely academic; it is the single greatest barrier to accurate risk assessment. Without understanding how NCM transforms and how its released elements interact biologically, any evaluation of population‐level risk is speculative at best. This uncertainty paralyzes our ability to develop effective safety regulations and threatens to undermine public trust in the very technologies meant to secure our future.

Here, we bridge this critical gap by establishing a complete, evidence‐based framework that connects the fundamental biology of NCM transformation to real‐world health risk assessment. We first reveal the sustained dissolution of NCM in a physiologically relevant lung environment, defining the precise multi‐metal mixture it generates. We then decipher the complex toxicological interactions within this biologically‐formed cocktail. Crucially, leveraging this mechanistic insight, we develop and validate the first quantitative risk assessment model capable of accurately capturing these interactions. By applying this model to real‐world exposure data, we uncover previously hidden, yet significant, population‐level health risks. Our work provides an essential, evidence‐based framework to ensure that the clean energy transition is not only green but, fundamentally, safe for human well‐being.

## 2 Results

### 2.1 Physicochemical Properties of Four NCM Cathode Materials

**FIGURE 1:**
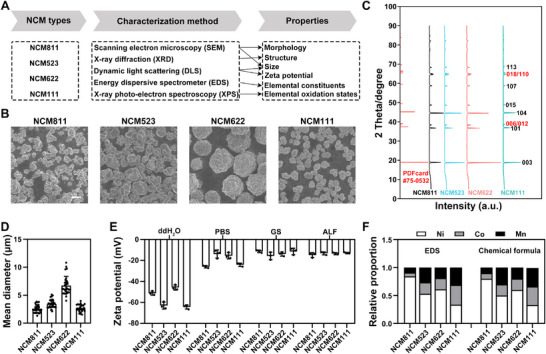
Physicochemical characterization of four NCM cathode materials. (A) Flowchart for physicochemical characterization of NCM. (B) Scanning electron microscopy (SEM) images of NCM. Scale bar = 5 µm. (C) X‐ray diffraction (XRD) patterns of NCM and the standard XRD pattern of hexagonal α‐NaFeO_2_‐type structure with an R3m space group. (D) Quantification of the mean diameter of NCM from SEM. n = 30. (E) Zeta potential of NCM in ddH_2_O, phosphate buffer saline (PBS), Gamble's solution lung fluid (GS), and artificial lysosomal fluid (ALF). n = 3. (F) Quantification of the relative proportion of Ni, Co, and Mn elements in NCM from the energy dispersive spectrometer (EDS) and the relative proportion in the chemical formula. Data were expressed as mean ± SD.

The physicochemical properties of NCM811, NCM523, NCM622 and NCM111 were characterized (Figure 1A). Micrographs from SEM depicted NCM811 and NCM523 as single‐crystal particles with well‐defined edges and corners, and they exhibited a smooth surface (Figure 1B). Conversely, NCM622 and NCM111 were observed as spherical polycrystalline particles with a rough surface, composed of various nanoparticles. All NCM particles consistently exhibited similar positions of reflection peaks to the standard XRD pattern of a typical hexagonal α‐NaFeO_2_‐type structure, classified with an R3m space group (PDF card #75‐0532) (Figure 1C). Each NCM particle showed the significant cleavage of diffraction peaks at the positions of (006)/(102) and (018)/(110), suggesting that the particles maintained a stable layered structure [11]. Consistently, the ratio of c‐lattice parameter to a‐lattice parameter (i.e., *c/a*) was larger than 4.899 (i.e., the standard *c/a* value of ideal cubic close packing), indicating that their layered structure was well‐arranged (Table S1). Moreover, the ratio of *I*(003)/*I*(104), which serves as an indicator of the cation mixing extent for Ni^2+^ occupying the site of Li^+^, displayed the highest ratio for NCM622, followed by NCM111, NCM811, and NCM523 (Table S1).

All NCM particles, except for NCM622, exhibited an average diameter of approximately 2–3 µm, and NCM622 showed an average diameter of approximately 6 µm (Figure 1D). The hydrodynamic diameters of NCM particles had a similar distribution between ddH_2_O, phosphate buffer saline (PBS), Gamble's solution lung fluid (GS), and alveolar lysosomal fluid (ALF) (Figure S1A–D), which was approximately the diameters obtained from SEM (Figure 1D). All NCM particles possessed a similar negative zeta potential in PBS, GS, and ALF, and a more negative potential in ddH_2_O (Figure 1E). Further elemental constituents from EDS revealed the presence of O, Ni, Co, and Mn elements in the NCM particles (Figure S1E). Ni, Co, and Mn were uniformly distributed on the surface of the material, while element O consistently exhibited the highest atomic percentage across all NCM particles. The atomic ratios of Ni:Co:Mn were found to be 0.84:0.07:0.09 for NCM811, 0.52:0.21:0.27 for NCM523, 0.61:0.20:0.19 for NCM622, and 0.34:0.35:0.31 for NCM111 (Figure 1F), aligning precisely with their nominal stoichiometries. XPS was employed to investigate the oxidation states of the transition metals. Given that the reference binding energy of Ni^2+^ (853.8 eV) and Ni^3+^ (855.8 eV) at Ni 2p_3/2_ [13], NCM811 (855.70 eV) showed the highest Ni^3+^ content and consequently the lowest Ni^2+^ content, followed by NCM622 (855.66 eV), NCM523 (854.99 eV), and NCM111 (855.18 eV) (Figure S2). All four NCM materials contained Co^3+^, as evidenced by their Co 2p_3/2_ peaks located at approximately 780.36–780.71 eV. The Mn 2p_3/2_ peaks, observed at similar binding energies (642.45–642.66 eV), suggested that Mn^4+^ was the dominant oxidation state in all samples.

### 2.2 Sustained Biological Dissolution of NCM in Physiologically Relevant Fluids

**FIGURE 2:**
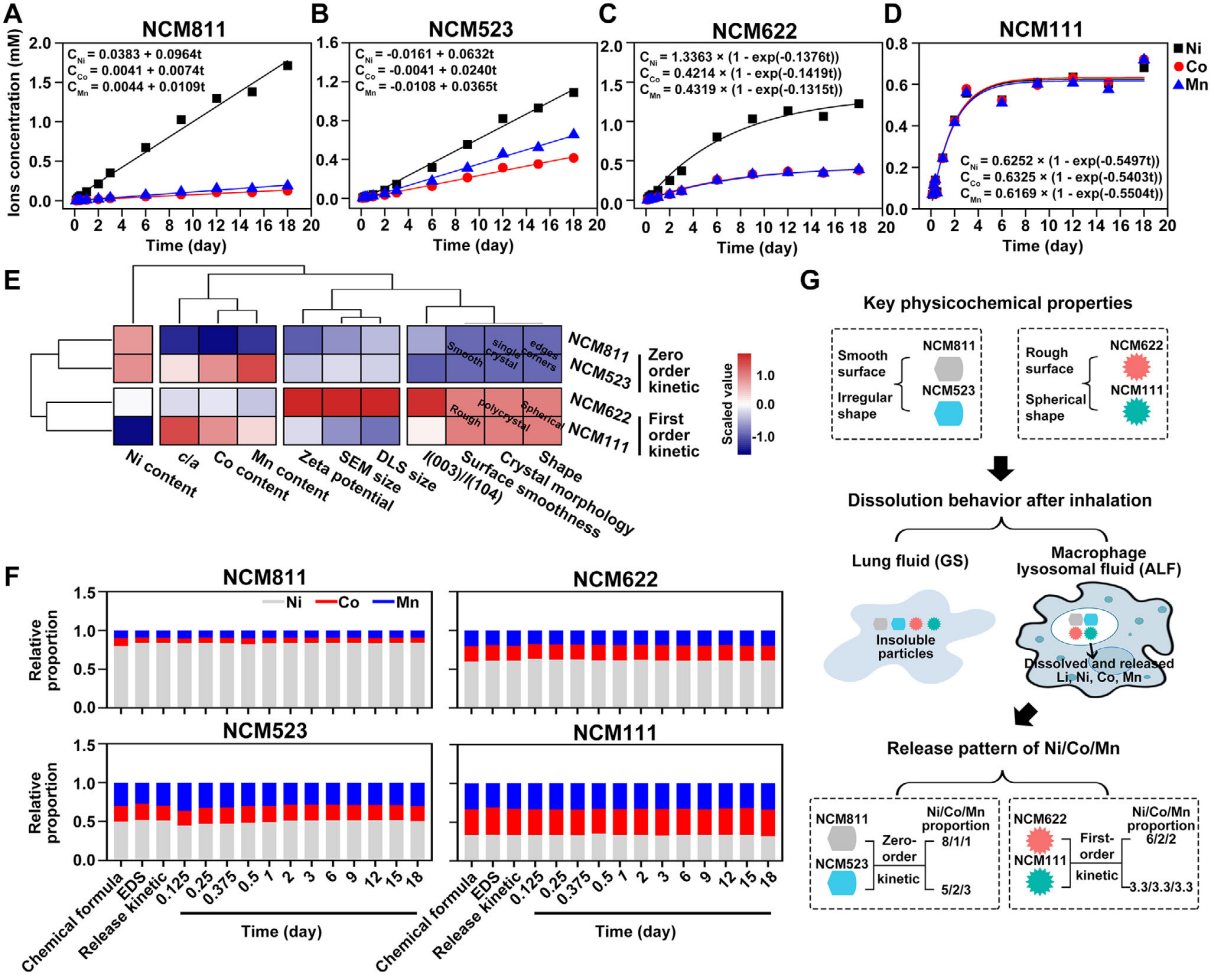
Sustained dissolution of NCM particles in simulated lysosomal fluid reveals distinct release kinetics for Ni, Co, and Mn. Concentrations of released Ni, Co, and Mn from (A) NCM811 and (B) NCM523 in ALF, illustrating the zero‐order release kinetics represented by the equation C_t_ = C_0_ + *k*t. Concentrations of released Ni, Co, and Mn from (C) NCM622 and (D) NCM111 in ALF, presenting the first‐order release kinetics described by the equation C_t_ = C_max_ × (1–exp(‐*k*t)). (E) Cluster analysis between the element release kinetics in ALF and the physicochemical properties of NCM particles. (F) The relative proportion of Ni, Co, and Mn from the chemical formula, EDS assessment, the value of *k* in the release kinetics, and released elements from NCM811 and NCM523, and the value of C_max_ in the release kinetics, and released elements from NCM622 and NCM111. (G) Schematic diagram illustrating the dissolution behavior of NCM particles upon inhalation into the lung and the different release patterns of Ni, Co, and Mn from NCM particles influenced by their key physicochemical properties.

We evaluated the biodegradation profile of NCM materials by assessing their stability and elemental release behaviors in multiple simulated physiological fluids to elucidate the potential risk‐inducing forms associated with inhalation exposure. NCM particles showed minimal release of Li, Ni, Co, and Mn in ddH_2_O, PBS, and GS, remaining largely intact over 18 days (Figure S3A). However, in ALF, an obvious dissolution of elements was detected in 3 h, and the concentrations of these elements significantly increased over time (Figure S3B), indicating that ALF may aid in particle degradation. All four types of NCM particles exhibited a time‐dependent increase of released elements during the 18 days period, with final concentrations of Ni, Co, and Mn similar to those expected for complete dissolution (Table S2), suggesting full digestion in lysosomes. Further evaluation of their element release kinetics showed that NCM811 and NCM523 particles exhibited a fixed release rate constant (i.e., the value of *k*) for Ni, Co, and Mn (Figure 2A,B), indicating zero‐order kinetics. In contrast, the release of Ni, Co, and Mn from NCM622 and NCM111 particles was initially rapid but gradually decelerated (Figure 2C,D), aligning with first‐order kinetics. The release pattern of Li from all NCM particles followed the same first‐order kinetics as Ni, Co, and Mn from NCM622 and NCM111 (Figure S4). We then investigated the potential reasons for the varying release kinetics between NCM (Figure 2E; Figure S5). Clustering analysis revealed that the release kinetics might be influenced by crystal morphology, shape, and surface smoothness (Figure 2E), with a significant Spearman correlation coefficient of 1.00 (*p*< 0.01, Figure S5). Specifically, NCM811 and NCM523, with well‐defined edges, smooth surfaces, and single‐crystal particles, exhibited a zero‐order kinetic release pattern for Ni, Co, and Mn (Figure 2E). In contrast, spherical NCM622 and NCM111, with rough surfaces and polycrystalline particles, displayed a first‐order kinetic release pattern (Figure 2E).

Knowing the elemental composition and proportion of the rapidly released element mixtures from NCM is an important prerequisite for further accurate evaluation of its risk. For NCM811, the relative proportions of Ni, Co, and Mn remained stable at each time point (Figure 2F). More importantly, the relative proportions of released Ni, Co, and Mn were similar to the proportions determined by EDS and the chemical formula (Figure 2F). Similarly, the other NCM particles maintained a consistent relative proportion of Ni, Co, and Mn at all time points, and matched their proportions from EDS and the chemical formula (Figure 2F). Overall, the original relative proportions of Ni, Co, and Mn in NCM particles determined their proportions in the released mixtures over time.

Once internalized by a cell with phagocytic function, like alveolar macrophages (AMs), the particles are metabolized in lysosomes, releasing various elements and acting on a wide range of cells [14]. Our findings illustrate that NCM particles inhaled into the lung are rarely dissolved in lung fluids but are instead internalized by cells with phagocytic function, such as AMs, and digested within lysosomes, releasing Ni, Co, and Mn mixtures in proportions mirroring their original proportions in the particles (Figure 2G). Given that particles of 2–3 µm rarely penetrate biological barriers (Figure 1D), the rapid dissolution of NCM represents a previously unappreciated pathway for systemic health risks via the released elemental mixtures.

### 2.3 The Biologically Generated Metal Mixture Induces Mitochondrial Toxicity

**FIGURE 3:**
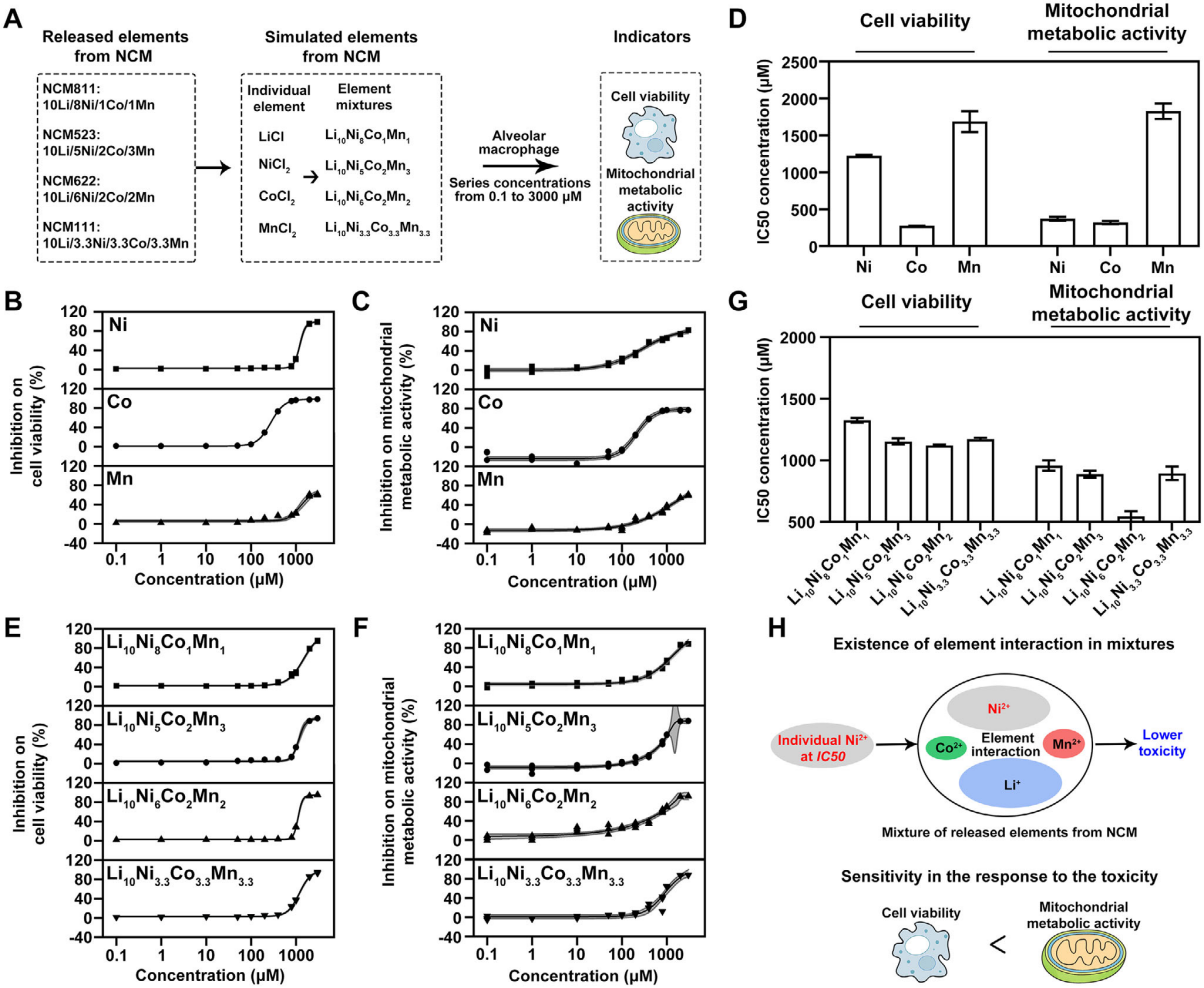
Effects of individual element and element mixture on both cell viability and mitochondrial metabolic activity. (A) Schematic diagram of evaluating the toxicity of elements released from four NCM particles on cell viability and mitochondrial metabolic activity using simulated elements and element mixtures based on the aforementioned compositions. The toxicity of individual Ni^2+^, Co^2+^, and Mn^2+^ on (B) cell viability (n = 3), (C) mitochondrial metabolic activity (n = 3), and (D) related half maximal inhibitory concentration (*IC50*) concentrations. Data were shown in *IC50* ± standard error (SE). The toxicity of simulated element mixtures on (E) cell viability (n = 3), (F) mitochondrial metabolic activity (n = 3), and (G) related *IC50* concentrations. Data were shown in *IC50* ± SE. (H) Schematic diagram illustrating the existence of element interactions in the toxicity of element mixture from four NCM particles and the greater sensitivity of mitochondrial metabolic activity.

We then simulated elemental mixtures representative of the compositional patterns identified in the particle release profiles, using the bivalent states of Ni, Co, and Mn to explore the potential toxicity of elemental mixtures released from NCM (Figure 3A). Understanding the toxicity of a single element is an important basis for further analyzing the interaction involved in the mixture, identifying the dominant factors in toxicity, and toxicity prediction. Specifically, Li showed no effect on cell viability or mitochondrial metabolic activity (Figure S6A,B). In contrast, Ni, Co, or Mn all exhibited concentration‐dependent inhibition on both cell viability (Figure 3B) and mitochondrial metabolic activity (Figure 3C), suggesting they might be the main source of mixture toxicity. Moreover, Ni, Co, and Mn induced different toxicity according to their respective half maximal inhibitory concentration (*IC50*) concentrations on cell viability and mitochondrial activity (Table S3), in which a lower *IC50* concentration suggests a higher toxicity [15]. For the toxicity on cell viability, Co exhibited the lowest *IC50* concentration (276.0699 µM), followed by Ni (1224.9724 µM) and Mn (1687.3019 µM) (Figure 3D). A similar trend of the *IC50* concentrations on mitochondrial metabolic activity was observed, which Co posed the lowest *IC50* (321.7393 µM), followed by Ni (372.3090 µM) and Mn (1828.5483 µM) (Figure 3D).

Further assessment of the element mixtures exhibited clear concentration‐dependent inhibition on both cell viability and mitochondrial metabolic activity (Figure 3E,F; Figure S6C). Mixture Li_10_Ni_6_Co_2_Mn_2_ showed the lowest *IC50* concentrations for cell viability (1120.7464 µM) and mitochondrial metabolic activity (543.1419 µM) among four mixtures (Figure 3G; Table S3), suggesting its highest toxicity. Compared to the *IC50* of mixture Li_10_Ni_6_Co_2_Mn_2_, a sequential increase in *IC50* from Li_10_Ni_5_Co_2_Mn_3_, Li_10_Ni_3.3_Co_3.3_Mn_3.3_, and Li_10_Ni_8_Co_1_Mn_1_ was observed for cell viability (1152.6603, 1171.7649, and 1325.5172 µM) and mitochondrial metabolic activity (887.2415, 893.7998, and 957.2196 µM) (Figure 3G; Table S3). Interestingly, our findings revealed that the *IC50* for Ni alone (372.3090 µM) was lower than that of mixtures containing Ni at the *IC50* for mixtures (Table S3). This shows that Ni in the mixture could cause greater toxicity if it acted alone, but the existence of other elements interfered with its toxicity, leading to the decrease of the overall toxicity of the mixture, which may be due to the antagonistic interactions between elements.

In summary, our results suggest that element mixtures from different NCMs may possess diverse toxicity on the cell viability and mitochondrial metabolic activity (Figure 3H). More importantly, we revealed a potential interaction between elements in mixtures, leading to decreased toxicity compared to single elements. Considering the varied proportion of Ni, Co, and Mn in element mixtures from different NCMs, the varied mixture toxicity might be caused by different element interactions. Moreover, the toxicity assessment of individual elements and element mixtures revealed a consistent pattern in both cell viability and mitochondrial metabolic activity. However, a higher *IC50* concentration was observed for mitochondrial metabolic activity in each element and element mixture (Figure S7A). Therefore, mitochondrial metabolic activity exhibited a more sensitive response to the toxicity of the element mixtures from NCM (Figure 3H), which could be primarily concerned.

### 2.4 Deciphering the Antagonistic‐Synergistic Interplay Within the Metal Mixture

**FIGURE 4:**
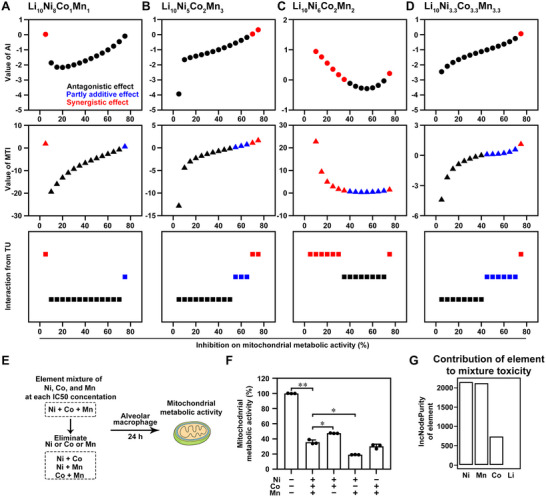
Deciphering the antagonistic‐synergistic interplay of Ni, Co, and Mn in the NCM‐derived metal mixture. Interaction analysis of the elements in (A) Li_10_Ni_8_Co_1_Mn_1_, (B) Li_10_Ni_5_Co_2_Mn_3_, (C) Li_10_Ni_6_Co_2_Mn_2_, and (D) Li_10_Ni_3.3_Co_3.3_Mn_3.3_ using additive index (AI), mixture toxicity index (MTI), and toxic unit (TU). Antagonistic effect (AI< 0, MTI< 0) was shown in black, synergistic effect (AI >0, MTI >1) was shown in red, and partly additive interaction (1 >MTI >0) was shown in blue. Detailed information on the AI, MTI, and TU was listed in Table S4. (E) Schematic diagram of evaluating the interaction among Ni^2+^, Co^2+^, and Mn^2+^ mitochondrial metabolic activity using binary and ternary element mixtures based on the *IC50* concentration. (F) The inhibition of Ni^2+^, Co^2+^, and Mn^2+^ binary and ternary element mixtures on mitochondrial metabolic activity. n = 3, ^*^ *p*< 0.05, ^**^ *p*< 0.01. Statistical analysis was performed using one‐way ANOVA followed by Dunnett's multiple comparisons test. The adjusted *p*‐value of control, Ni^2+^/Co^2+^, and Ni^2+^/Mn^2+^ vs. Ni^2+^/Co^2+^/Mn^2+^ group was 0.0014, 0.0367, and 0.0250, respectively. (G) The IncNodePurity of individual elements to mitochondrial metabolic activity of element mixtures released from four NCM particles.

We subsequently delve deeper into these complex interactions in element mixtures from NCM to support a more accurate risk evaluation of NCM by employing a range of classic mixture toxicity indices, including additive index (AI), mixture toxicity index (MTI), and toxic unit (TU) [16]. The indices revealed that the interactions among Ni, Co, and Mn in the Li_10_Ni_8_Co_1_Mn_1_ mixture exhibited a predominantly antagonistic effect (Figure 4A; Table S4). Li_10_Ni_5_Co_2_Mn_3_ mixture displayed less antagonistic and more additive and synergistic effects compared to Li_10_Ni_8_Co_1_Mn_1_ (Figure 4B; Table S4). Similarly, the Li_10_Ni_6_Co_2_Mn_2_ mixture showed even less antagonism and more additive and synergistic effects than Li_10_Ni_5_Co_2_Mn_3_ (Figure 4C; Table S4). For the Li_10_Ni_3.3_Co_3.3_Mn_3.3_ mixture, the antagonistic effect was less pronounced than in Li_10_Ni_8_Co_1_Mn_1_, but more than in Li_10_Ni_6_Co_2_Mn_2_ (Figure 4D; Table S4). The presence of antagonistic effects suggests that the elements in the mixtures mutually inhibit each other's toxicity, leading to a reduction in total toxicity [16]. Conversely, synergistic effects enhance individual toxicities, thereby increasing the toxicity of the mixture [16]. Therefore, the decreased antagonistic effects along with the increase of synergistic effects in Li_10_Ni_8_Co_1_Mn_1_, Li_10_Ni_3.3_Co_3.3_Mn_3.3_, Li_10_Ni_5_Co_2_Mn_3_, and Li_10_Ni_6_Co_2_Mn_2_ explain the differences in toxicity of element mixtures (Li_10_Ni_8_Co_1_Mn_1_< Li_10_Ni_3.3_Co_3.3_Mn_3.3_< Li_10_Ni_5_Co_2_Mn_3_< Li_10_Ni_6_Co_2_Mn_2_).

We further confirm the interactions between Ni, Mn, and Co in the element mixtures by eliminating the Ni, Co, or Mn from the ternary mixtures of these elements at each *IC50* concentration (Figure 4E). The comparison of the changes in mitochondrial metabolic activity showed that removal of Mn from the ternary mixtures reduced toxicity, suggesting that adding Mn enhances toxicity (Figure 4F). In contrast, the elimination of Co promoted the toxicity, and the elimination of Ni had no significant effect. This confirms that the addition of Co and Ni does not increase total toxicity but instead inhibits it, potentially due to their antagonistic effects on the toxicity of the ternary mixtures. Furthermore, eliminating similar proportions of Ni, Co, or Mn from simulated element mixtures derived from NCM particles also showed similar improvements in toxicity due to Mn and inhibition effects of Ni and Co on the toxicity of the element mixtures (Figure S7B,C).

We conducted a further analysis to determine the relative contribution of each element to the overall toxicity of their mixtures, using the IncNodePurity metric from a random forest algorithm [17]. Ni was identified as the most influential element, with the highest IncNodePurity, suggesting its pivotal role in the toxicity of the mixtures (Figure 4G). Mn also exhibited significant importance, similar to Ni, while Co had a lower IncNodePurity but still contributed to the toxicity. In contrast, Li's IncNodePurity was minimal, indicating its lesser role in mixture toxicity. Considering the key role of Ni in the toxicity of the mixtures and its inhibitory effect, and Mn's promoting effect on toxicity, these findings underscore the importance of focusing on these elements when developing new NCM materials. Ni's ability to lead in antagonistic effects and toxicity, and Mn's role in synergistic effects, highlight the significance of these elements in the toxicity of element mixtures from NCM particles.

### 2.5 The IAI Model: A New Framework for Accurate Risk Assessment of NCM‐Derived Metal Mixtures

**FIGURE 5:**
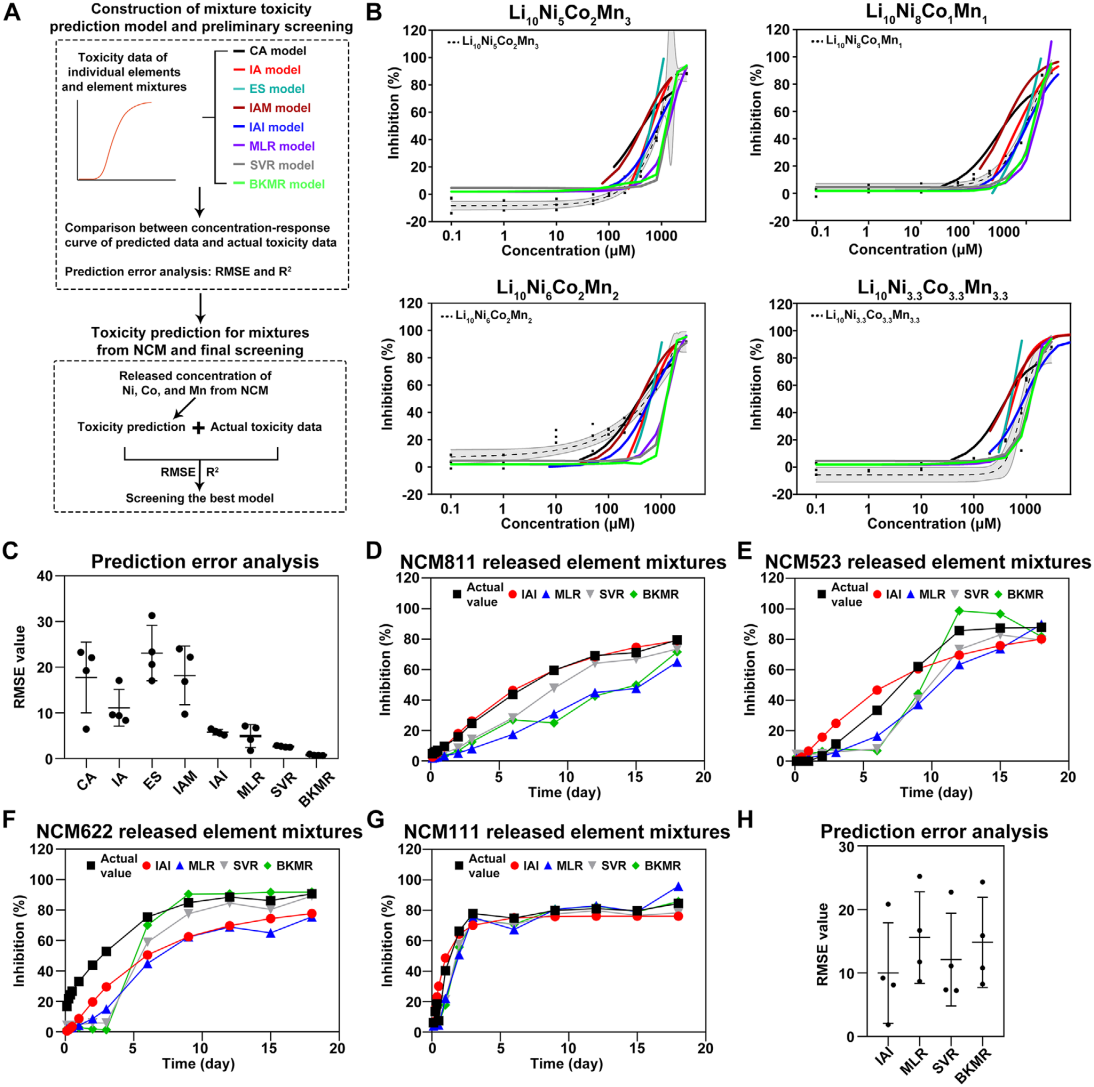
Screening identifies the IAI model as the optimal framework for predicting NCM mixture toxicity. (A) Schematic diagram of element mixtures toxicity prediction based on the toxicity data of simulated elements using concentration addition model (CA), independent action model (IA), effect summation model (ES), integrated addition model (IAM), the IAI, multiple linear regression (MLR), support vector regression (SVR), and Bayesian kernel machine regression (BKMR) model and comparison for the best toxicity prediction model based on the detected concentration of released elements from NCM particles. (B) The concentration‐response curve of Li_10_Ni_8_Co_1_Mn_1_, Li_10_Ni_5_Co_2_Mn_3_, Li_10_Ni_6_Co_2_Mn_2_, Li_10_Ni_3.3_Co_3.3_Mn_3.3,_ and the related concentration‐response curve from toxicity prediction models for mitochondrial metabolic activity toxicity. (C) Root mean square error (RMSE) value of the comparison of the toxicity from CA, ES, IA, IAM, IAI, MLR, SVR, and BKMR models with the toxicity of element mixtures. The actual value of inhibition on mitochondrial metabolic activity of element mixtures released from (D) NCM811, (E) NCM523, (F) NCM622, and (G) NCM111, based on the detected concentration of Ni, Co, and Mn released from four NCM particles, and related predictive value from the toxicity prediction model. (H) Comparison of the accuracy of the toxicity from IAI, MLR, SVR, and BKMR models with the actual value of toxicity based on the detected concentration of Ni, Co, and Mn released from four NCM particles.

We then established several quantitative toxicity assessment models and identified the optimal one capable of incorporating the complex interactions between Ni, Mn, and Co in the mixture to deduce the risk threshold for more accurate risk assessment of NCM. We first screened the most suitable toxicity assessment models for the four mentioned NCM, based on the current toxicity data of these NCM particles. Major models are employed, including concentration addition model (CA), independent action model (IA), effect summation model (ES), integrated addition model (IAM), integrated Addition and Interaction (IAI), support vector regression (SVR), and Bayesian kernel machine regression (BKMR). We first fitted these models based on the toxicity data of individual elements and simulated element mixtures (Figure 5A). To preliminarily select the most accurate models, we compared the model‐quantified toxicity data with the actual toxicity data of the element mixtures (Figure 5A). The models that best matched the actual concentration‐response curve were considered the most accurate. Specifically, the IA, IAI, MLR, SVR, and BKMR models showed better alignment with the actual curve compared to the CA, ES, and IAM models (Figure 5B), suggesting higher accuracy in toxicity quantification.

We further validated the accuracy of these models by comparing their quantified toxicity with the actual toxicity of the element mixture using R‐square (R^2^ and root mean square error (RMSE) (Figure 5C; Table S5). Models with lower RMSE and higher R^2^ were deemed more accurate. The RMSE values of IAI, MLR, SVR, and BKMR models were lower than that in the other models, proving that they have better quantification accuracy. The higher R^2^ of the IAI, MLR, SVR, and BKMR model than the other models also demonstrated their relatively good quantification ability (Figure S8A and Table S5).

The IAI, MLR, SVR, and BKMR models were then further screened using the actual element mixtures released from NCM particles to identify the best model for toxicity quantification of NCM (Figure 5A). Compared to the toxicity of the element mixtures released from NCM811, the quantified toxicity from the IAI model was closest (Figure 5D), suggesting a better performance of the IAI model. Similarly, the toxicity quantified by the IAI model was closest to the toxicity of element mixtures released from NCM523 (Figure 5E). Regarding the toxicity of released element mixtures from NCM622, the quantified toxicity from BKMR might be closest to the actual values (Figure 5F). All four models showed similar predictions for the toxicity of element mixtures from NCM111 (Figure 5G). Final comprehensive comparison revealed that the IAI model had the lowest RMSE among the four models (Figure 5H), indicating its highest ability to quantify the toxicity of element mixtures released from NCM particles, which was also supported by its highest R^2^ (Figure S8B). Collectively, we developed and screened out an IAI model to quantify the toxicity and deduce the risk threshold of element mixtures released from NCM particles.

### 2.6 Significant Population‐Level Health Risks Revealed by the New Assessment Framework

**FIGURE 6:**
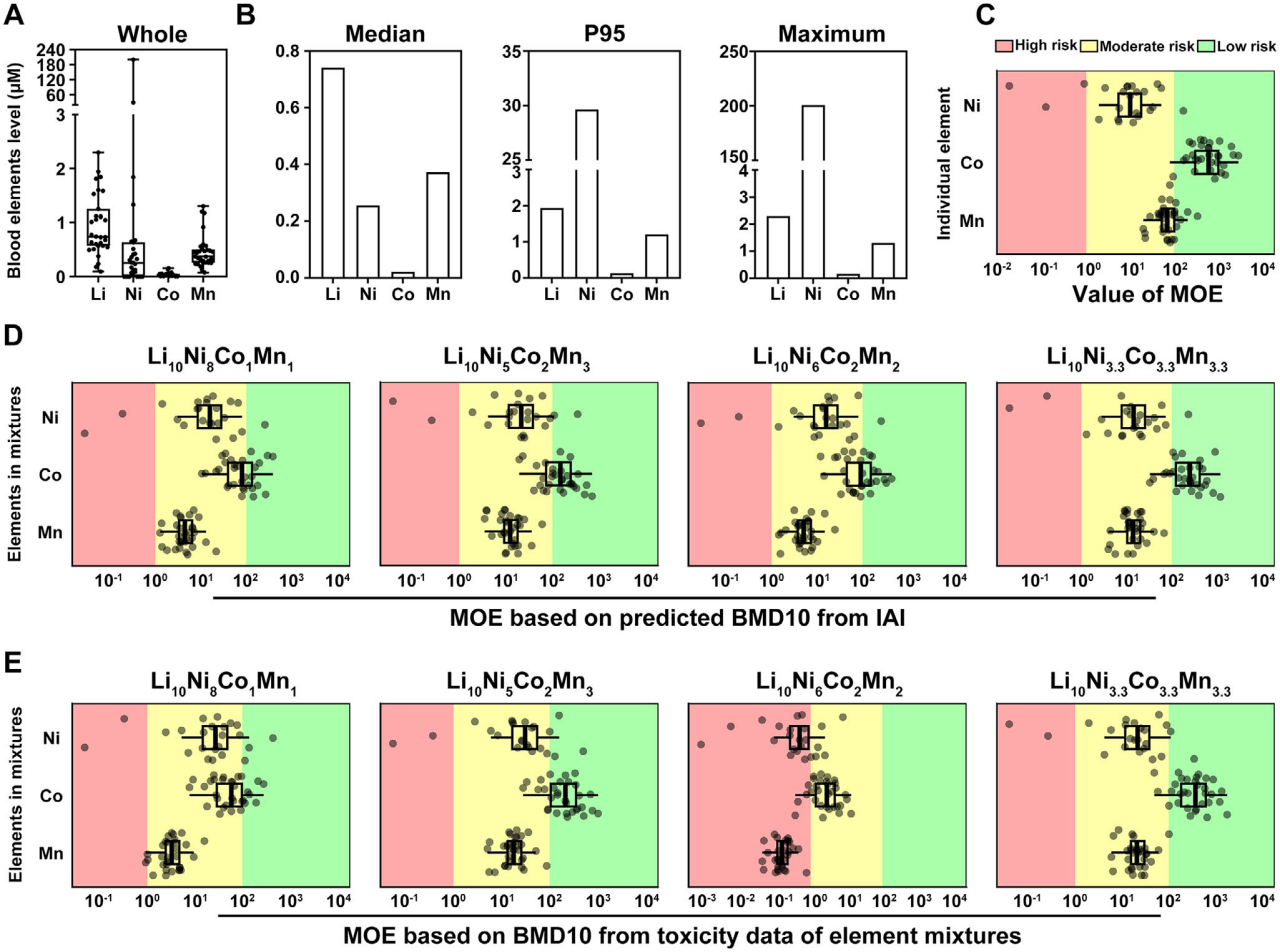
Real‐world risk assessment using the IAI model reveals significant health risks in highly exposed populations. (A) The blood levels of elements in all participants. n = 24–31. (B) The median, 95th percentiles (P95), and maximum level of the element in the population. Values of margin of the exposure (MOE) according to the *IC10* concentrations from the mitochondrial metabolic activity toxicity of (C) individual elements, and IAI models of (D) Li_10_Ni_8_Co_1_Mn_1_, Li_10_Ni_5_Co_2_Mn_3_, Li_10_Ni_6_Co_2_Mn_2_, and Li_10_Ni_3.3_Co_3.3_Mn_3.3_ and the level of Ni, Co, and Mn in the blood from the population related to NCM particles production. (E) Values of MOE according to the *IC10* concentrations from experiment data of Li_10_Ni_8_Co_1_Mn_1_, Li_10_Ni_5_Co_2_Mn_3_, Li_10_Ni_6_Co_2_Mn_2_, and Li_10_Ni_3.3_Co_3.3_Mn_3.3_. MOE ≤ 1 means high risks, 1< MOE ≤ 100 means moderate risks, and 100< MOE means low risks.

Followed by the construction and screening of the optimal model to deduce the risk threshold for risk assessment, we utilized it to assess the health risks for the population in the NCM particle manufacturing industry with high exposure to NCM. We first assessed the internal exposure levels of Li, Ni, Co, and Mn in the blood of these workers. Li, Ni, and Mn were found to have higher levels compared to Co (Figure 6A; Table S6). Li exhibited the highest median blood level (0.740 µM), followed by Mn (0.372 µM), Ni (0.256 µM), and Co (0.021 µM) (Figure 6B). The range of Ni level was broader than Li, Co, and Mn (Figure 6A), with the 95th percentiles (*P95*) level of Ni (29.643 µM) being higher than Li (1.942 µM), Mn (1.204 µM) and Co (0.133 µM), and the maximum levels followed a similar trend (C_Ni_ = 200.596 µM, C_Li_ = 2.296 µM, C_Mn_ = 1.306 µM, C_Co_ = 0.158 µM) (Figure 6B), indicating higher exposures to Li, Ni and Mn compared to Co.

Margin of exposure (MOE), a representative health risk assessment approach [18], was applied to assess the health risks of NCM. The value of MOE represents the ratio of the dose that causes a small but measurable effect (i.e, the BMD10, equivalent to the *IC10* concentration in this study) to the actual exposure level [18]. We evaluated the MOE for each element in the NCM particle based on the *IC10* from the concentration‐response curve or calculated by the IAI model. Considering that Li did not show toxicity (Figure S6A,B), it was excluded from the MOE assessment. For single element exposure, the MOE for Ni and Mn were moderate (1< MOE< 100), while the risk for Co exposure was low (MOE >100) (Figure 6C). For element mixtures calculated by the IAI model, the risk from Co was around the border of moderate and low, while Ni and Mn still posed a moderate risk. Comparing the MOE calculated by the IAI model with that based on experimental *IC10* data, Co showed similar risk levels (Figure 6D). Similarly, moderate risks were observed for Ni and Mn based on experimental data (Figure 6E), confirming the reliability of the IAI model in assessing these risks. The potential health risks associated with high exposure to NCM particles emphasize the need for immediate health protection measures and regulatory policies for the relevant high‐exposed population.

## 3 Discussion

The promise of a sustainable energy transition is fundamentally challenged by a critical knowledge gap: the health risks of its core materials, like NCM cathodes, are poorly understood because our assessment paradigms ignore their biological fate. Our study directly confronts this challenge by establishing a new paradigm: the health hazard of NCM is dictated not by the particle, but by its dynamic biological transformation into a toxic metal cocktail. We reveal for the first time that inhaled NCM undergoes sustained lysosomal dissolution, releasing a multi‐metal mixture whose toxicity is governed by a unique interplay of antagonistic (Ni, Co) and synergistic (Mn) interactions. This fundamental discovery of NCM's biological fate provided the essential key to unlock accurate risk assessment. Building on this insight, we identified the IAI model as the only framework capable of capturing these complex elemental dynamics. Applying this model to real‐world exposure data uncovers significant, yet previously hidden, population‐level health risks. Our work provides the essential evidence‐based framework that connects in‐body material transformation to real‐world health outcomes, a critical step toward ensuring that the clean energy transition is not only green but fundamentally safe for human well‐being.

Our findings demonstrate that the primary health risk of inhaled NCM originates from its sustained dissolution within the acidic environment of alveolar macrophage lysosomes. We observed that NCM particles remain largely inert in simulated lung fluids but are rapidly metabolized once phagocytosed [19, 20], consistent with their particle size and the central role of macrophages in clearance [21]. Dissolution kinetics depend critically on the physical morphology. Single‐crystal materials (NCM811, NCM523) exhibited zero‐order kinetics (constant release rate), while polycrystalline materials (NCM622, NCM111) followed first‐order kinetics (concentration‐dependent rate) [22]. This distinction may arise because polycrystalline particles possess a larger specific surface area and chemically active grain boundaries that accelerate degradation [23, 24]. Specifically, single‐crystal particles have smaller specific surface areas due to their smooth surface [23], leading to fewer active interfaces. Moreover, its dense structure leads to the reaction rate being governed by the surface chemical reaction rate [25]. Conversely, polycrystalline particles have rougher surfaces with grain boundaries that are highly reactive under acidic conditions, leading to rapid degradation [24, 26]. Consequently, polycrystalline particles rapidly release substantial amounts of the elemental mixture early on, generating higher exposure concentrations that induce greater toxicity. These findings indicate the critical role of NCM particle morphology and structure in dissolution behavior and post‐inhalation hazards. Crucially, regardless of the kinetic profile, the relative proportions of released metals consistently mirrored the stoichiometry of the parent NCM material (Table S7), revealing a direct link between battery composition and the hazardous metal mixture generated in the body. To reconcile environmental differences between simulated lysosomal fluid and cellular lysosomes, we monitored pH changes during NCM incubation. Simulated lysosomal fluid maintained pH stability (∼4.0) over 18 days, confirming a sustained driving force for metal release (Figure S9A). Although intracellular lysosomal pH in macrophages peaked at day 1 and returned to baseline by days 2–6 following NCM exposure (Figure S9B,C). The persistence of this acidic milieu indicates a continuous driving force for metal release from NCM. These results support the facilitation of NCM dissolution by an acidic environment, but further research is warranted to investigate dissolution kinetics using primary macrophages and in vivo models to account for complex physiological variables.

The biological transformation of NCM actively creates a distinct toxicological entity. We found that the acute mitochondrial toxicity of NCM particles is almost entirely recapitulated by their simulated ion mixtures (Figure S10), indicating that rapid metal release is the primary driver of initial toxicity. Moreover, mixture toxicity is not merely additive but governed by complex elemental interactions (Figure 3H). We deciphered a unique signature where Ni^2+^ and Co^2+^ antagonistically suppress toxicity, while Mn^2^^+^ acts as a potent synergist. Mn^2+^ catalyzes Fenton‐like reactions [27, 28], potentiating oxidative stress when coexisting with pro‐oxidative metals (e.g., Ni^2^^+^, Co^2^^+^) [29]. Consistent with this, adding Mn^2+^ to Ni^2+^/Co^2+^ mixtures significantly increases ROS levels, whereas reverse addition does not alter or reduce ROS (Figure S11A,B). This aligns with Mn^2+^ exacerbating the inhibition of mitochondrial metabolic activity (Figure 4F). Notably, this synergy appears mediated through metabolic disruption rather than membrane depolarization, as no concordant changes in mitochondrial potential were observed (Figure S11C,D). Furthermore, Mn^2+^ may amplify toxicity by perturbing the phospholipid bilayer structure [30, 31], increasing membrane permeability, and facilitating the uptake of co‐existing metals. Conversely, Ni^2+^ and Co^2+^ antagonism likely stems from competitive uptake. As divalent cations with similar ionic radii, they share transporters (e.g., DMT1, ZIP14) [32, 33], reducing bioavailability as one limits the internalization of the other [34]. Intracellular competition of Ni^2+^ and Co^2+^ for binding proteins and molecular targets [35] decreases effective target occupancy, explaining the observed attenuation of mixture toxicity. This mechanistic insight (Figure S12) is the cornerstone of our risk assessment approach. Traditional models fail to capture such dynamics [36, 37]. The IAI model, by incorporating both individual toxicities and interaction coefficients, proved uniquely capable of accurately quantifying the risk [37]. This establishes a direct, causal chain: from material morphology, to dissolution kinetics, to elemental interactions, and finally, to a predictive, quantitative risk model.

Since its proposal [36, 37], the IAI model has served as a promising approach for assessing mixture toxicity [38, 39]. A primary advantage of the IAI model over classical models (i.e., CA and IA) is its ability to account for component interactions. While CA assumes simple additive toxicity and IA treats constituents as independent events, both neglect interactions within the mixture [40]. The IAI model builds upon an additive framework and explicitly incorporates interactions via the K‐function [41], contributing to its higher reliability. In this study, we calculated the necessary parameters (i.e., IC50_Ni_, IC50_Co_, IC50_Mn_, K_Ni_, K_Co_, and K_Mn_). This allows the IAI model to be broadly applied to predict toxicity and assess risks for emerging NCM particles due to their similar constituent elements (Li, Ni, Co, and Mn). Moreover, the IAI model's predictive validity is generally confined to mixtures with constituents similar to those used for its calibration. For other heterometallic material (i.e., LiFePO_4_), application requires detailed dose‐response data for each component and the mixture to determine the dose‐response slope (p) and interaction parameters (K) [37]. This reliance on experimental data means that experimental errors can propagate and amplify the model's uncertainty. Additionally, the application of the IAI model is constrained by the concentration range of the training data. At extreme concentrations, saturation of toxic effects or the activation of distinct mechanisms may compromise prediction accuracy [42]. The model's utility is also endpoint‐dependent; therefore, caution is advised when extrapolating to outcomes such as oxidative stress or inflammation, which may involve different sensitivities or mechanisms. Furthermore, variations in metabolic kinetics across different exposure routes (e.g., inhalation vs. oral) necessitate a careful evaluation of toxicokinetic assumptions before applying the IAI model to diverse scenarios [43]. Nevertheless, the superior predictive performance of the IAI model demonstrated in our study underscores its potential as a valuable tool for the risk assessment of technologically critical material mixtures.

Selecting an appropriate and sensitive endpoint is critical in risk assessment. In this study, we compared cytotoxicity, a classical outcome in risk assessment [44], with mitochondrial metabolic activity, a process fundamental to macrophage function [45]. Mitochondrial inhibition proved to be a more sensitive indicator of cellular stress (Figure S7A). This aligns with substantial evidence establishing mitochondrial dysfunction as a critical link between exposure and health impairments and a pivotal player in disease pathogenesis [46, 47, 48, 49]. Given this sensitivity and biological relevance, we selected mitochondrial metabolic inhibition to derive the BMD10. The BMD approach is a cornerstone of next‐generation risk assessment, having largely superseded traditional metrics such as LD50 or NOAEL/LOAEL [50]. Its superiority lies in detecting low‐dose, early biological effects and minimizing model uncertainty by incorporating full dose‐response data [51]. Therefore, the BMD10 for mitochondrial metabolic inhibition provided the most robust and scientifically defensible Point of Departure (POD) for our risk assessment.

The ultimate validation of any risk assessment paradigm lies in its applicability to real‐world scenarios. In cases such as battery recycling, exposure often involves complex mixtures including anode materials (e.g., graphite), separators (e.g., polyethylene), and additives (e.g., organic phosphorus flame retardants) [52, 53]. While such mixtures reflect reality, studying single pollutants remains a critical prerequisite for systematically deconstructing combined risks. This approach isolates the effects of NCM from the confounding variables of other battery components, enabling the establishment of clear causal relationships. Moreover, our risk assessment framework is grounded in exposure data from an occupational cohort of NCM production workers—a group primarily exposed to the pure active substance. This alignment provides a unique opportunity to validate our experimental findings against direct human evidence. By applying our IAI‐derived risk thresholds to exposure data from workers in the battery manufacturing industry, we uncovered significant, moderate health risks that were previously hidden. The observed blood metal levels in these workers—highest for Li, followed by Ni and Mn, and lowest for Co—align remarkably well with the composition of prevalent NCM materials and known toxicokinetic pathways (e.g., rapid renal clearance of Co vs. pulmonary retention of Ni) [54‐ [58]. This concordance serves as a powerful validation of our entire framework, confirming that the biological fate and elemental interactions we identified in vitro are directly relevant to human exposure.

For the human health risk assessment in this study, the in vitro BMD10 derived from mouse cells was extrapolated to a human equivalent dose using a body surface area‐based conversion factor. We acknowledge inherent uncertainties, as this approach may not fully capture complex inter‐species differences in toxicokinetics and toxicodynamics or intra‐species variability [59]. Future refinements could involve sensitivity analyses, human‐relevant models (e.g., organoids), and physiologically based toxicokinetic (PBTK) models to better simulate the absorption, distribution, metabolism, and excretion (ADME) of the test substances [60]. Nevertheless, to address these uncertainties, we conducted a detailed analysis incorporating variability in both toxicological benchmarks and exposure levels (Figures S13 and S14 and Table S8). The distribution of the MOE was stable following the inclusion of uncertainties from both BMD10 and exposure data (Figure 6C–E; Figures S13 and S14). This consistency confirms that our risk conclusions remain robust despite the inherent variability. Our work provides the definitive evidence needed to move from scientific speculation to evidence‐based regulation, offering a clear roadmap for policymakers to protect workforce health and maintain public trust in the clean energy transition.

Our study proposes a novel framework for understanding NCM particle transformation and toxicity, and identifies key directions for future research to connect fundamental science, industrial applications, and public policy. While commercial NCM materials are suitable for elucidating specific mechanisms, real cathode black mass typically contains additional components, such as PVDF binders, conductive carbon, and residual electrolytes [61]. These components modify surface properties and dissolution kinetics, potentially altering biotransformation and toxicity. Future studies using authentic black mass should incorporate these complexities to enhance the translational relevance of the proposed risk assessment framework. At the mechanistic level, a critical next step is to elucidate the links between particle dissolution kinetics and physicochemical properties, thereby guiding the rational design of safer materials. Additionally, future work should unravel the precise molecular pathways governing the observed antagonistic‐synergistic interplay, particularly how Mn potentiates toxicity at the cellular level. Future in vivo studies are crucial to map the full ADME profile of NCM materials and to evaluate chronic, multi‐organ impacts to address the full spectrum of human health risks. By pursuing these avenues, we can ensure that the transition to a sustainable future is not only green and efficient but, above all, fundamentally safe and just for humanity.

In conclusion, our study provides the critical scientific foundation to resolve a central paradox of the clean energy transition: how to harness new materials without compromising human health. As summarized in Figure S15, we establish a transformative paradigm that shifts the focus from the static particle to the dynamic biological fate of the material. By revealing the sustained lysosomal dissolution of NCM and deciphering the antagonistic‐synergistic interplay of its released metals, we unlocked the ‘black box’ of its toxicity. This mechanistic insight was instrumental in developing the IAI model, the first framework capable of accurately translating these complex biological interactions into quantitative risk. The application of this model to real‐world data provides the unequivocal evidence needed to move from speculation to regulation. Ultimately, our work ensures that the pursuit of a sustainable planet is inextricably linked to the protection of human health. It provides a roadmap for policymakers and industry to navigate the energy transition, guaranteeing that the technologies of our future are not only green and efficient but, above all, fundamentally safe for humanity.

## 4 Experimental Section

### 4.1 Materials and Solution Preparation

NCM with compositions LiNi_0.8_Co_0.1_Mn_0.1_O_2_ (NCM811, CAS 179802‐95‐0), LiNi_0.6_Co_0.2_Mn_0.2_O_2_ (NCM622, CAS 193215‐05‐3), LiNi_0.5_Co_0.2_Mn_0.3_O_2_ (NCM523, CAS 193215‐53‐1), and LiNi_1/3_Co_1/3_Mn_1/3_O_2_ (NCM111, CAS 346417‐97‐8) were purchased from Guangdong Canrd New Energy Technology Co., Ltd (Guangdong, China). Lithium chloride (LiCl, 99.5 %, L573611), nickel chloride hexahydrate (NiCl_2_·6H_2_O, 99.9 %, N118563), and manganese chloride tetrahydrate (MnCl_2_·4H_2_O, 99.9 %, M109463) were purchased from Aladdin Biochemical Technology Co., Ltd (Shanghai, China). Cobalt chloride hexahydrate (CoCl_2_·6H_2_O, 98.0 %, C8661) was purchased from Sigma–Aldrich Trading Co, Ltd (Shanghai, China). Stock solutions (50 mM) of LiCl, NiCl_2_, MnCl_2_, and CoCl_2_ were prepared by dissolving them in deionized water (ddH_2_O) and stored in darkness at 4°C. Simulated element mixture solutions were prepared to mimic the elemental ratios of Li/Ni/Co/Mn found in the NCM materials. The molar ratios were based on the respective chemical formulas: 10/8/1/1 for NCM811, 10/6/2/2 for NCM622, 10/5/2/3 for NCM523, and 10/3.3/3.3/3.3 for NCM111. Consistent with the stoichiometry of these NCM compounds, the Li content was always equal to the sum of Ni, Co, and Mn. Consequently, the Li concentration was set as the total concentration for the mixed solutions, reflecting its stable proportion within the NCM particles. The working solution was freshly prepared by diluting the stock solution (50 mM) with cell culture medium before usage.

### 4.2 Characterization of NCM

For scanning electron microscope (SEM) analysis, 2 mg of NCM particles were dispersed in anhydrous ethanol and sonicated for 5 mins. A 1–2 drop aliquot of the suspension was pipetted onto conductive adhesive carbon tape mounted on an aluminum SEM sample holder. The morphology and particle size of NCM were assessed using a scanning electron microscope (SU8000, Hitachi, Tokyo, Japan) operated at an accelerating voltage of 3 kV, a magnification of 2000×, and a working distance of 8.3 mm. Mean diameters of NCM were quantified by measuring 30 randomly selected particles from SEM images using Image J software (Version 1.8.0, https://imagej.net/ij/) according to the scale bar. The X‐ray diffraction (XRD) with Cu Kα radiation at 40 kV and 40 mA in the 2θ range from 5° to 90° was used to investigate the crystal structure of all NCM samples on a PANalytical X'Pert PRO (PANalytical, Netherlands). The hydrodynamic radius and zeta potentials (ζ) of NCM suspensions (200 µg/mL) in ddH_2_O, PBS, GS, and ALF were detected using dynamic light scattering (DLS) with a Zeta‐Sizer (Malvern, UK).

Elemental surface composition of NCM, including oxygen (O), Ni, Co, and Mn, was determined using an XFlash FlatQuad energy dispersive X‐ray spectroscopy (EDS) detector system (Bruker Nano GmbH Berlin, Germany). Samples were analyzed at a magnification of 22 000×, a high voltage of 15 keV, and a working distance of 0.45 mm. Elemental mapping and micrographs were used for analysis, and the normalized atomic percentages of O, Ni, Co, and Mn were calculated. The oxidation states of transition metals in NCM were analyzed by X‐ray photoelectron spectroscopy (XPS; ULVAC‐PHI PHI5000). Experimental parameters were as follows: 2–6 scans, Al Kα X‐ray source, spot size of 500 µm, standard lens mode, constant analysis energy (CAE) mode with a pass energy of 40.0 eV, and an energy step size of 0.050 eV. The energy scale was calibrated using the C1s peak at 284.8 eV. Data analysis was performed using Avantage software (v5.52, Thermo Fisher Scientific, USA). All measurements were performed in triplicate for each condition.

### 4.3 Determination of Released Elements From NCM Particles in ddH_2_O, PBS, GS, and ALF

The release of elements (Li, Ni, Co, Mn) from NCM particles was quantified under different physiological and aqueous conditions: ddH_2_O, PBS, GS, and ALF. The specific compositions and concentrations of GS and ALF, based on a previous study [62], were detailed in Tables S9 and S10, respectively. Stock suspensions of NCM particles at a concentration of 200 µg/mL were prepared in each test medium (ddH_2_O, PBS, GS, ALF). For each condition, 30 mL of the respective suspension was transferred into a sealed 50 mL glass conical flask [63]. All flasks were incubated on a thermostatic orbital shaker maintained at 37°C and 150 rpm [64]. The total incubation period was set to 18 days, a duration chosen to align with the half‐life of alveolar macrophages [65], the primary phagocytic cells involved in clearing inhaled particles. Sampling was performed at designated time points (3 h, 6 h, 9 h, 12 h, 1 d, 2 d, 3 d, 6 d, 9 d, 12 d, 15 d, and 18 d). At each interval, 2 mL of the supernatant was carefully collected from a flask. The collected samples were centrifuged at 14000 *g* for 20 min to pellet residual particles. The resulting supernatant was then diluted to a final volume of 10 mL using 1 % nitric acid (HNO_3_; SINOPHARM, Beijing, China). Following filtration through a 0.45 µm water‐based membrane filter, prepared samples were analyzed via Inductively Coupled Plasma‐Optical Emission Spectrometry (ICP‐OES) (Optima 7000DV, PerkinElmer, Massachusetts, USA) to detect the concentrations of released Li (λ = 670.784 nm), Ni (λ = 231.604 nm), Co (λ = 228.620 nm), and Mn (λ = 257.610 nm). Detailed operating parameters for the ICP‐OES instrument are listed in Table S11. Quantification of element concentrations was achieved by referencing standard curves generated from a mixed Li, Ni, Co, and Mn (GNM‐M25213‐2013, Guobiao (Beijing) Testing & Certification Co., Ltd, Beijing, China) at concentrations of 0, 0.01, 0.05, 0.1, 0.5, 1, and 5 mg/L.

### 4.4 Construction of Element Release Kinetic Curves

To elucidate the release kinetics of Li, Ni, Co, and Mn from NCM particles, kinetic curves were constructed by plotting the measured element concentrations against time. This analysis was performed using Origin 2021 (version 9.8.0.200). The experimental data were fitted to two common kinetic models: the zero‐order kinetic model and the first‐order kinetic model. The zero‐order kinetics indicated a steady release rate during the release process, while the release rate in the first kinetic is proportional to the NCM concentration. Based on the equation of the zero‐order kinetic (C_t_ = C_0_ + *k*t) and first‐order kinetic (C_t_ = C_max_ × (1–exp(‐*k*t))), the time was set as an independent variable, and the corresponding element concentration was set as a dependent variable to fit the equation of zero‐order kinetic and first‐order kinetic, respectively. C_t_ represents the released concentration at time. C_0_ is the initial element concentration in solution. *k* is the corresponding release rate constant. And C_max_ represents the maximum concentration of elements released into the solution.

### 4.5 Correlation and Cluster Analysis of Physicochemical Properties and Release Kinetics

To explore the relationships between NCM particle physicochemical properties and the kinetics of Li, Ni, Co, and Mn release, we performed correlation and cluster analyses using R statistical software (Version 4.0.0) via RStudio. Prior to analysis, both physicochemical property data and release kinetic data were normalized using zero‐one standardization. Spearman's rank correlation coefficient was then calculated to assess the monotonic relationships between these variables, and the resulting correlation matrix was visualized using the corrplot package (Version 0.92). Hierarchical clustering was subsequently performed. The resulting cluster relationships were visualized as a heatmap using the pheatmap package (Version 1.0.12).

### 4.6 Cell Culture and Viability Assessment

MH‐S cells, a murine alveolar macrophage‐like cell line with correct short tandem repeat (STR) identification (https://shoposs.servicebio.cn/2024/10/29/1730194220911ubkIaC.pdf), were purchased from Wuhan Servicebio Technology Co., Ltd. on November 26th, 2024 (STCC20007P, Wuhan, China). Cells were maintained in RPMI 1640 medium (BasalMedia, Shanghai, China), supplemented with 10 % fetal bovine serum (FBS, Boster, Pleasanton, CA, USA), 100 U/mL penicillin, and 0.1 mg/mL streptomycin (meilunbio, Dalian, China), in a humidified incubator at 37°C and 5 % CO_2_. Cell viability was assessed by 7‐aminoactinomycin D (7‐AAD, 51–68981E, BD Biosciences, New Jersey, USA), a dye that selectively stains non‐viable cells by binding to DNA after penetrating compromised cell membranes [66]. MH‐S cells were plated in 6‐well plates at a density of 6 × 10^5^ cells/well and allowed to adhere overnight. Cells were then treated for 24 h with a range of concentrations (0, 0.1, 1, 10, 50, 100, 200, 400, 800, 1000, 2000, and 3000 µM) of individual salts (LiCl, NiCl_2_, CoCl_2_, MnCl_2_) or simulated mixtures thereof, mimicking elemental release from NCM particle (composition details provided in Table S12). Following treatment, cells were harvested, washed twice with PBS, and incubated with 0.5 µM 7‐AAD for 15 min at 37°C. After another PBS wash, cells were resuspended in 600 µL PBS with 1 % FBS. The fluorescence of 7‐AAD was detected by flow cytometry (CytoFLEX, Beckman Coulter, CA, USA) at the excitation and emission wavelengths (E_x_/E_m_ = 545 nm/650 nm). The proportion of dead cells, identified as the 7‐AAD^+^ population, was quantified by FlowJo_v10.6.2 (BD Life Sciences, USA).

### 4.7 Mitochondrial Metabolic Activity Assessment

Alamar blue assay, a high‐throughput and sensitive method, was employed to evaluate the mitochondrial metabolic activity. The assay measures changes in fluorescence intensity, which correlate with the activity of mitochondrial enzymes involved in NADH/NAD, NADPH/NADP, FADH/FAD, and FMNH/FMN redox processes within the TCA cycle and oxidative phosphorylation [67, 68]. MH‐S cells were plated in 96‐well plates at a density of 2 × 10^4^ cells/well and allowed to adhere overnight. Cells were then treated for 24 h under the following conditions. For the single element and simulated element mixtures treatment, the cells were stimulated with a concentration series (0, 0.1, 1, 10, 50, 100, 200, 400, 800, 1000, 2000, and 3000 µM) of LiCl, NiCl_2_, CoCl_2_, MnCl_2_ individually, or to simulated element mixtures derived from NCM particles (using the chloride salts at the same concentrations). For the evaluation of potential interactions among Ni, Co and Mn, cells were treated with simulated mixtures containing reduced proportions of Ni, or Co, or Mn, based on their *IC50* concentrations (details composition in Table S13). For the NCM particles treatment, cells were exposed to 200 µg/mL of NCM811, NCM523, and NCM111. Following the treatment, cells were washed twice with PBS. Subsequently, cells were incubated with 100 µL culture medium containing 10 µL Alamar Blue reagent (C7084, Bioss, Beijing, China) for 1 h in the dark at 37°C. Fluorescence was detected using a microplate reader (Thermo Fisher Scientific, USA) at 530 nm/590 nm (E_x_/E_m_). All experiments were conducted in triplicate. Relative mitochondrial metabolic activity inhibition was calculated using the formula: Inhibition on mitochondrial metabolic activity (% of control) = 100–(Fluorescence of treated sample / Fluorescence of control sample) × 100.

### 4.8 Construction of Concentration‐Response Curves

The experimental data of inhibition on cell viability and mitochondrial metabolic activity were fitted using the non‐linear least squares method with Origin 2021 (version 9.8.0.200) software to obtain the concentration‐response curves. For each data set, the model with the best fit was selected based on the R^2^ and RMSE. Specifically, the Lorentz, Logistic, and Logistic5 equations were selected for the concentration‐response curves.

The Lorentz equation:

(1) $$\mathrm{IR}=IR_{\max}+\frac{2A}{\pi }\frac{w}{4{\left(c-EC_{50}\right)}^{2}+w^{2}}$$

The Logistic equation:

(2) $$\mathrm{IR}=\frac{IR_{\min}-IR_{\max}}{1+{\left(\frac{c}{EC_{50}}\right)}^{p}}+IR_{\max}$$

The Logistic5 equation:

(3) $$\mathrm{IR}=IR_{\min}+\frac{IR_{\max}-IR_{\min}}{{\left(1+{\left(\frac{EC_{50}}{c}\right)}^{h}\right)}^{s}}$$

Wherein, *IR* is the inhibition rate on cell viability and mitochondrial metabolic activity, *IR_max_* is the maximum inhibition rate, *A* is the area under the concentration‐response curves, *w* is the full width at half maximum, *c* is the concentration, *IR_min_* is the minimum inhibition rate, *EC50* is the concentration at which the response reached 50% of the effect range between the lower and upper effects, *p* and *h* are the slope of the curve, and *s* determines the symmetry of the curve. The details of each parameter were listed in Tables S14–S16.

### 4.9 Importance Evaluation of Element in Mixtures

To evaluate the importance of the individual element within element mixtures contributing to the toxicity, the IncNodePurity index from the random forest model was applied. This index considered the overall contribution of each element to the prediction accuracy of the model. The prediction error of the model will increase if an element is removed or its value is randomly shuffled, with the larger the increase, the more important the element. Therefore, higher index values of IncNodePurity indicate greater importance of the individual element. The model was built using the R package of “randomForest (4.7–1.1)” and the IncNodePurity index was measured according to the total reduction in node impurities from splits on each variable, averaged across all trees.

### 4.10 Interaction Analysis of Elements in Mixtures

The interaction of elements in the toxicity of element mixtures was analyzed by three classic mixture toxicity indexes [16], including AI, MTI, and TU, according to previous studies [69, 70]. TU, AI, and MTI can be estimated using Formulas (4), (5), and (6), respectively:

(4) $$TU_{i}=\frac{c_{i}}{IC50_{,i}},M=\sum_{i=1}^{n}TU_{i},M_{0}=\frac{M}{TU_{max}}\;$$

*c_i_* is the concentration of the *i^th^* element under the specified effect, and *IC50_, i_* is the concentration of the *i^th^* element provoking 50 % IR. If *M*< 1, it indicates a synergy effect; if *M* = 1, it suggests an additive effect; if *M_0_* >*M* >1, it indicates a partial additive effect; if *M* = *M_0_*, it suggests an independence effect; and if *M* >*M_0_*, it indicates an antagonistic effect.

(5) $$AI=\left\{\begin{array}{c} 1/M-1,M\leq 1\\ 1-M,M>1 \end{array}\right.\;$$

If *AI* >0, it indicates a synergy effect; if *AI* = 0, it suggests an additive effect; if *AI*< 0, it indicates an antagonistic effect.

(6) $$MTI=1-\frac{\log M}{\log M_{0}}$$

If *MTI* >1, it indicates a synergy effect; if *MTI* = 1, it suggests an additive effect; if 1>*MTI* >0, it indicates a partial additive effect; if *MTI* = 0, it suggests an independence effect; if *MTI*< 0, it indicates an antagonistic effect.

### 4.11 Toxicity Prediction of Element Mixtures

Prediction of element mixtures was applied by five joint effects models (i.e., CA, IA, ES, IAM, and IAI) [37, 38, 39], and three machine learning models (i.e., MLR using the built‐in “lm()” function in R, SVR using the R package of “e1071 (1.7–9)”, and BKMR using the R package of “bkmr (0.2.2)”).

Based on the CA model, the total concentration of elements in an element mixture provoking *x* % effect (*IC_x,mixture_*) can be calculated using the following formula:

(7) $$p_{i}=\frac{c_{i}}{c_{mixture}}$$

(8) $$IC_{x,mixture}={\left(\sum_{i=1}^{n}\frac{p_{i}}{IC_{x,i}}\right)}^{-1}\;$$

According to the IA model, the total toxicity of element mixtures is obtained by the product of the fractional toxicity of individual elements:

(9) $$IR\;\left(c_{mixture}\right)=\left(1-\prod_{i=1}^{n}\left[1-\frac{IR\left(c_{i}\right)}{100}\right]\right)\;\times 100$$

In the ES model, the total toxicity of element mixtures is obtained by the summation of the fractional toxicity of individual elements:

(10) $$IR\;\left(c_{mixture}\right)=\sum_{i=1}^{n}IR\left(c_{i}\right)\;$$

The IAM model is combined with the CA and IA models, and the total toxicity of element mixtures is calculated using the following formula:

(11) $$IR\;\left(c_{mixture}\right)=\frac{100}{1+\frac{1}{{\left(\sum_{i=1}^{n}\frac{c_{i}}{IC_{50,i}}\right)}^{p^{\prime }}}}\;$$

The IAI model extends the IAM model by incorporating the *K* function to account for interactions. The total toxicity of element mixtures is calculated using the following formula:

(12) $$K_{i}=\frac{IC_{50,i}}{IC_{50,mixture}}\;$$

(13) $$IR\;\left(c_{mixture}\right)=\frac{100}{1+\frac{1}{{\left(\sum_{i=1}^{n}\frac{K_{i}\times c_{i}}{IC_{50,i}}\right)}^{p^{\prime }}}}$$

*p_i_* is the ratio of the *i^th^* element relative to the elements in the element mixture, *IC_x,mixture_* is the concentration of the element mixture at *x* % *IR, IC_x,i_* is the concentration of the *i^th^* element at *x* % *IR* solely, *IR(c_i_)* is the individual effect of the *i^th^* element if present in the concentration *c_i_*, *IR(c_mixture_)* is the joint effect of the element mixture with the total concentration *c_mixture_*, *p'* is the average slope of the individual element within an element mixture, and *K_i_* is a function that describes the influence of the *i^th^* element on the total toxicity. The details of each parameter for IAI model in this study were listed in Table S17.

Machine learning models were constructed based on the concentration of each element according to the proportion of elements in the element mixtures. The training set consisted of toxicity experimental data of the element mixtures at different concentrations in concentration‐response curve fitting. The validation set included the actual released concentration of individual elements at various time points from NCM particles and the corresponding mitochondrial toxicity data.

### 4.12 Accuracy Evaluation of Toxicity Prediction Model

R^2^ and RMSE were used to evaluate the stability and accuracy of the toxicity prediction model. A smaller RMSE and a larger R^2^ indicated a smaller prediction error and better model performance. R^2^ and RMSE were calculated using Formulas (14) and (15), respectively.

(14) $$R^{2}=1-\frac{\sum_{i=1}^{n}{\left(I^{\prime }C_{i}-IC_{i}\right)}^{2}}{\sum_{i=1}^{n}{\left(IC_{i}-\bar{IC}\right)}^{2}}$$

(15) $$RMSE=\sqrt{\frac{1}{n}\sum_{i=1}^{n}{\left(IC_{i}-I^{\prime }C_{i}\right)}^{2}}\;$$

*n* represents the number of molecules used in the model prediction; *IC_i_* and $\overset{\prime }{\underset{i}{IC}}$ represent the actual IC values from experiments and calculated IC values, respectively; and $\bar{IC}$ represents the average of the true actual IC values from experiments of all the calculated molecules.

### 4.13 MOE Analysis for Occupational Population

A cohort of 31 individuals (Table S18 for demographics) employed in NCM particle production for over 1 year, involved in tasks such as batching, centrifugation, operation of an automated line furnace, jet milling, quality testing, and packaging, was recruited. Whole blood samples (0.2 mL) were collected for internal exposure assessment of Li, Ni, Co, and Mn using ICP‐MS (ICP‐RQ, Thermo Fisher Scientific, USA) as previously described [71]. Samples were digested with 0.4 mL HNO_3_ in a microwave system (program: 100 W/5 min ramp, 10 min hold; 150 W/5 min ramp, 10 min hold; cool down) and diluted to 6 mL. Analysis was performed post‐filtration (0.44 µm) using instrument parameters: RF power = 1548.6 W, interface temperature = 31.2°C, cooling water flow rate = 0.41 L/min, cooling gas flow rate = 14.0 L/min, collision airflow = 4.9 mL/min, auxiliary gas flow rate = 0.80 L/min, atomization gas flow rate = 0.97 L/min, sampling depth = 15 mm, peristaltic pump speed = 40.0 rpm, scanning frequency = 3, and detector mode = KED. Internal standards and background correction were applied. Recovery ranged from 82.76 % ± 12.12 % to 107.32 % ± 7.92 %. Each sample was triplicated, with the average reported. The linear range for Li, Ni, Co, and Mn is 0–10 µg/L, with an R^2^ of 0.9999. The limits of detection (LOD) for Li, Ni, Co, and Mn are 0.06, 0.03, 0.021, and 0.048 µg/L, respectively. And the limits of quantification (LOQ) for Li, Ni, Co, and Mn are 0.2, 0.1, 0.07, and 0.16 µg/L, respectively. The details of detected Li, Ni, Co, and Mn concentrations (µM) in whole blood are listed in Table S19. This study obtained approval from the Ethics Committee of the Medical College of Qingdao University (QDU‐HEC‐2024296).

The MOE was calculated as the ratio of the benchmark dose at 10 % (BMD10), converted from mouse to human using a factor of 12 (based on body surface area), to the measured internal exposure levels in the study population. The BMD10 (equivalent to IC10) for individual Ni, Co, and Mn was derived from their respective concentration‐response curves. For the element mixture analysis, the BMD10 was based on the IC10 of the mixture, scaled according to the individual element proportions within the mixture. Similarly, for the IAI model, the MOE for Ni, Co, and Mn were derived using their concentrations within the mixture's IC10 as calculated by the IAI model, again applying the respective element proportions.

### 4.14 Statistical Analysis

Statistical analyses were performed using GraphPad Prism (version 9.1.0), R statistical software (Version 4.0.0) via RStudio, and Origin 2021 (version 9.8.0.200). Except for the data of element release from NCM, all of the experimental results were presented as mean ± standard deviation (SD) with at least three biological triplicates. One‐way analysis of variance (ANOVA) followed by Dunnett's multiple comparisons test was used to compare the significant differences between various groups. For the *p*‐values, ^*^ *p*< 0.05, ^**^ *p*< 0.01 were marked as statistically significant.

## Author Contributions

Ze Zhang and Gan Miao contributed equally. Ze Zhang: Conceptualization, Investigation, Methodology, Data curation, Formal analysis, Visualization, Writing – original draft. Gan Miao: Conceptualization, Investigation, Methodology, Data curation, Formal analysis, Visualization. Xueyu Zhang: Formal analysis. Zhao Shu: Methodology. Dawei Lu: Methodology, Funding acquisition. Yujie Song: Formal analysis. Shanfa Yu: Funding acquisition. Qian Liu: Writing – review and editing. Yang Song: Writing – review and editing. Rong Zhang: Funding acquisition. Xiaoting Jin: Writing – review and editing, Funding acquisition. Yuxin Zheng: Funding acquisition.

## Funding

This work was supported by grants from the National Natural Science Foundation of China (Grant numbers: 82241086, 82473674, 82473609, U24A20772, and 22222610), and the Qingdao Natural Science Foundation (24‐4‐4‐zrjj‐157‐jch).

## Inclusion and Ethics

This study obtained approval from the Ethics Committee Medical College of Qingdao University (QDU‐HEC‐2024296).

## Conflicts of Interest

The authors declare no conflict of interest.

## Supporting information




**Supporting File**: advs74102‐sup‐0001‐SuppMat.docx.

## Data Availability

All data supporting the findings of this study are available within the article and its supporting information file or from the authors upon reasonable request.
