# Supplementary material for: Redefining the Health Risk of Battery Materials Through a Biologically Transformed Metal Mixture
Source: Adv Sci (Weinh). 2026 Jan 30;13(19):e23469. doi: 10.1002/advs.202523469 (PMC13045502; doi:10.1002/advs.202523469)
Supplement: Supplementary file 1 — Supporting File: advs74102‐sup‐0001‐SuppMat.docx. [file ADVS-13-e23469-s001.docx]

Redefining the Health Risk of Battery Materials Through a Biologically Transformed Metal Mixture

Ze Zhang,^1†^ Gan Miao,^1†^ Xueyu Zhang,^1^ Zhao Shu,^2^ Dawei Lu,^2^ Yujie Song,^1^ Shanfa Yu,^3^ Qian Liu,^2^ Yang Song,^2^ Rong Zhang,^4^ Xiaoting Jin,^1*^ Yuxin Zheng^1*^

^1^ Department of Occupational Health and Environmental Health, School of Public Health, Qingdao University, Qingdao, 266071, China

^2^ State Key Laboratory of Environmental Chemistry and Ecotoxicology, Research Center for Eco-Environmental Sciences, Chinese Academy of Sciences, Beijing, 100085, China

^3^ Department of Public Health, Henan Medical College, Zhengzhou, 451191, China

^4^ Department of Toxicology, School of Public Health, Hebei Medical University, Shijiazhuang, 050017, China

^†^These authors share co-first authorship.

^*^These authors share co-senior authorship.

Address correspondence to Xiaoting Jin, School of Public Health, Qingdao University, Qingdao, China. Email: xtjin@qdu.edu.cn. And, Yuxin Zheng, School of Public Health, Qingdao University, Qingdao, China. Email: yxzheng@qdu.edu.cn.

**Table of Contents:**

**Supplemental Method**

Intracellular lysosomal pH assessment

Intracellular reactive oxygen species (ROS) level assessment

Dysfunctional mitochondria percent analysis

Uncertainty analysis of MOE

**Supplemental Figures and Legends**

**Figure S1.** Hydrodynamic diameter and elemental constituents of NCM particles.

**Figure S2.** XPS spectra of NCM.

**Figure S3.** Release of elements from NCM particles over 18 days.

**Figure S4.** Release pattern of Li from NCM particles characterized by the first-order kinetic.

**Figure S5.** Correlation analysis between the element release kinetics in ALF and physicochemical properties of NCM particles.

**Figure S6.** Inhibition on the cell viability and mitochondrial metabolic activity of individual element and element mixtures from NCM particles.

**Figure S7.** Comparison of the *IC50* concentration between cell viability and mitochondrial metabolic activity and inhibition of element mixtures on mitochondrial metabolic activity.

**Figure S8.** R^2^ value of toxicity from toxicity prediction model and the actual value of toxicity.

**Figure S9.** Changes of pH in alveolar lysosomal fluid and cellular lysosomes during NCM treatment.

**Figure S10.** Toxicity of NCM particles and simulated released element mixtures.

**Figure S11.** The effect of Ni, Co and Mn binary and ternary element mixtures on ROS level and dysfunctional mitochondria.

**Figure S12.** A schematic figure for the potential mechanism of Ni/Co antagonism and Mn synergy in mixture toxicity in this study.

**Figure S13.** Uncertainty analysis of population risk related to NCM particle production based on individual elements.

**Figure S14.** Uncertainty analysis of population risk related to NCM particle production based on NCM element mixtures.

**Figure S15.** Risk assessment of NCM exposure in population with high exposure level on the basis of NCM biological transformation-derived ion mixtures and involved ion-ion interactions.

**Supplemental Tables**

**Table S1.** Lattice parameters of NCM particles from XRD assay.

**Table S2.** Calculated concentration for complete dissolution and detected concentrations of released Li, Ni, Co, and Mn from NCM in the ALF after 18 days of incubation.

**Table S3.** Value of *IC50* for individual elements and element mixtures on mitochondrial metabolic activity.

**Table S4.** Calculation of AI, MTI, and TU for evaluating the interactions of individual elements in the mixtures.

**Table S5.** The RMSE and R^2^ of model-calculated toxicity and the actual toxicity of the element mixture.

**Table S6.** Levels of Li, Ni, Co and Mn in the blood of occupational populations exposed to NCM particles.

**Table S7.** Release parameter for Ni, Co, and Mn released from NCM particles.

**Table S8.** Uncertainty analysis of population risk related to NCM particle production.

**Table S9.** Components and relevant concentrations in GS.

**Table S10.** Components and relevant concentrations in ALF.

**Table S11.** Parameters and measurement conditions of ICP-OES.

**Table S12.** Experimental design for the mixture system of Li, Ni, Co, and Mn.

**Table S13.** Experimental design for the mixture system of Li, Ni, Co, and Mn at *IC50* concentration with reduced proportion of Ni, or Co, or Mn.

**Table S14.** Concentration-response curves parameters of Lorentz.

**Table S15.** Concentration-response curves parameters of Logistic.

**Table S16.** Concentration-response curves parameters of Logistic5.

**Table S17.** IAI model parameters.

**Table S18.** Demographic and characteristics of population cohort (*n* = 31).

**Table S19.** Concentrations of Li, Ni, Co, and Mn (μM) in whole blood.

**Intracellular lysosomal pH assessment**

The effect of NCM exposure on lysosomal pH of was analyzed using the LysoSensor Green DND-189 probe (MB6043, Meilunbio, Dalian, China). After exposure to 200 μg/mL NCM811 and NCM111 for 1, 2, 3, 6 days, the cells in 12-well plates were collected. Followed by incubation with 1 μM LysoSensor Green DND-189 for 30 min at 37°C the cells were washed twice with PBS, resuspended in 600 μL of PBS containing 1% FBS, and detected by flow cytometry at wavelengths according to the manufacturer's instructions. The mean fluorescence intensity of LysoSensor Green DND-189 was quantified by FlowJo_v10.6.22.

**Intracellular reactive oxygen species (ROS) level assessment**

The ROS level was analyzed using the DCFH-DA probe (HY-D0940, MedChemExpress, Shanghai, China). After exposure to ternary mixtures of Ni, Co, and Mn, as well as binary mixtures of Ni and Co, Ni and Mn, and Co and Mn at each corresponded IC50 concentration for mitochondrial metabolic activity (372.3090 μM for Ni, 321.7393 μM for Co, 1828.5483 μM for Mn), the cells in 12-well plates were incubated with 10 μM DCFH-DA for 30 min at 37°C. Following incubation, the cells were washed twice with PBS, resuspended in 600 μL of PBS containing 1% FBS, and detected by flow cytometry at the excitation (492 nm) and emission (517 nm) wavelengths according to the manufacturer's instructions. The mean fluorescence intensity of ROS was quantified by FlowJo_v10.6.22.

**Dysfunctional mitochondria percent analysis**

The accumulation of MitoTracker Red CMXRos (MB6046, Meilunbio, Dalian, China) in mitochondria depends on the mitochondrial membrane potential, while the ability of MitoTracker Green FM (MB6044, Meilunbio, Dalian, China) to localize to mitochondria is not affected by mitochondrial membrane potential and can label all mitochondria.^[^*^1^*^]^ Therefore, the MitoTracker Green^+^/MitoTracker Red^-^ cells indicate impaired mitochondrial function in the gated cells. After exposure to ternary mixtures of Ni, Co, and Mn, as well as binary mixtures of Ni and Co, Ni and Mn, and Co and Mn at each corresponded IC50 concentration for mitochondrial metabolic activity (372.3090 μM for Ni, 321.7393 μM for Co, 1828.5483 μM for Mn), the cells in 12-well plates were incubated with 500 nM MitoTracker Red CMXRos and 200 nM MitoTracker Green FM for 30 min at 37°C. The cells were then washed with PBS twice, resuspended in 600 μl PBS containing 1% FBS, and analyzed by flow cytometry (CytoFLEX, Beckman Coulter, CA, USA) at wavelengths according to the manufacturer's instructions. The percent of MitoTracker Green^+^/MitoTracker Red^-^ cells for each sample were quantified by FlowJo_v10.6.22.

**Uncertainty analysis of MOE**

An uncertainty analysis was conducted to characterize population variability and parameter uncertainty in the MOE estimates. Benchmark dose values at the 10% response level (BMD10) for individual elements and element mixtures were treated as uncertain parameters and assumed to follow log-normal distributions. For all BMD10 values, a common coefficient of variation (CV) of 0.5 was applied. The corresponding log-normal parameters (μ and σ) were derived from the reported point estimates to ensure consistency with the specified CV. Population exposure levels of Ni, Co, and Mn were derived from measured blood concentrations in populations related to NCM particle production. Exposure distributions were also assumed to be log-normally distributed, with distribution parameters estimated from the empirical mean and standard deviation of the observed data.

Monte Carlo simulations (n = 10,000 iterations) were performed to propagate uncertainties in both BMD10 and exposure estimates. In each iteration, MOE values were calculated as the ratio of simulated BMD10 to the corresponding simulated exposure concentration. This procedure generated probabilistic MOE distributions for each element and mixture scenario. From the simulated MOE distributions, key summary metrics were extracted, including the median, 2.5th and 97.5th percentiles, and the cumulative probabilities of MOE values falling below 100 and 1. The uncertainty results were visualized using cumulative probability plots on a logarithmic MOE scale, with reference lines indicating MOE = 1 and MOE = 100.


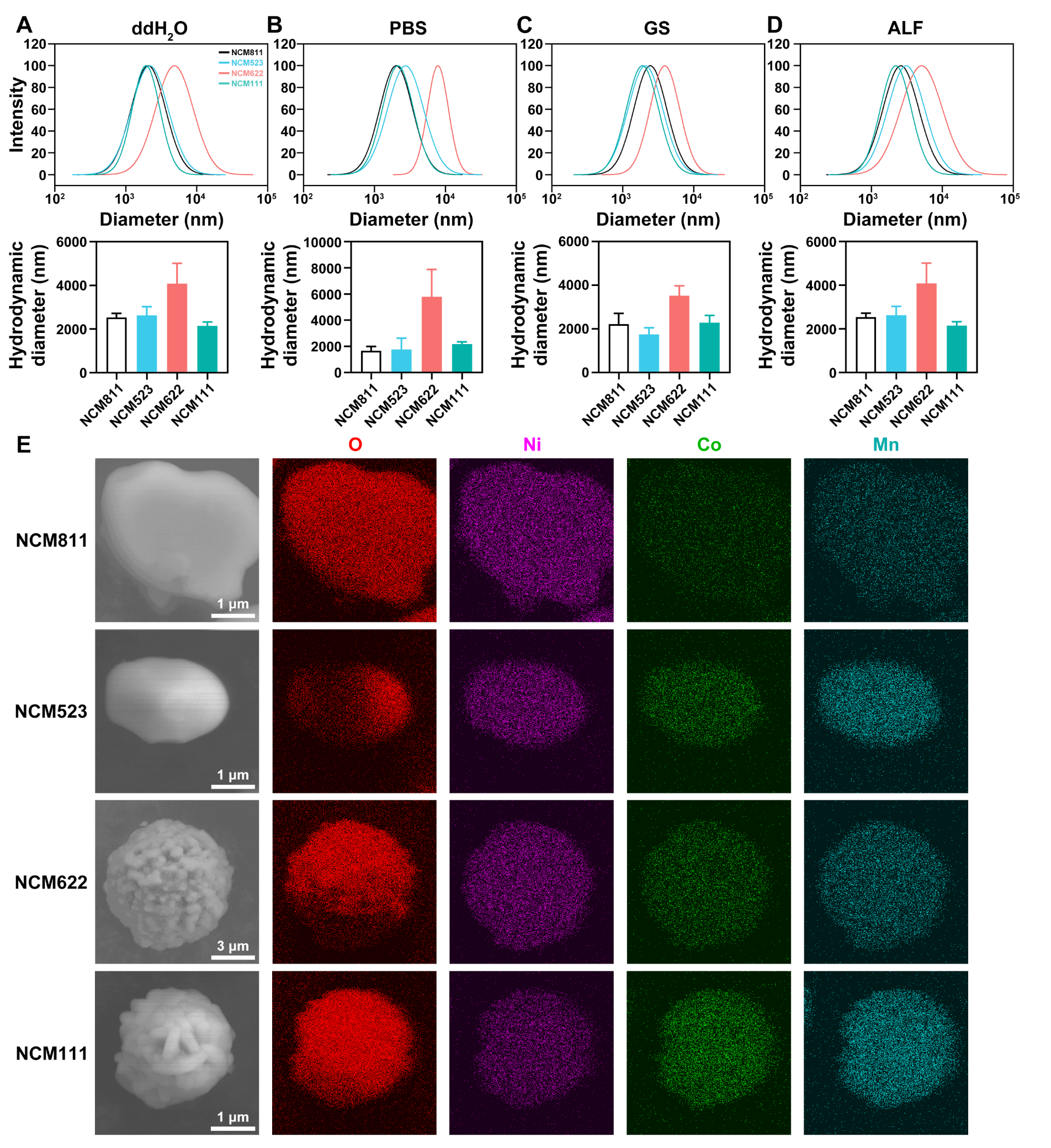


**Figure S1.** Hydrodynamic diameter and elemental constituents of NCM cathode materials. The distribution of hydrodynamic diameter and mean hydrodynamic diameter of NCM in (A) ddH_2_O, (B) phosphate buffer saline (PBS), (C) lung fluid (gamble’s solution lung fluid, GS), and (D) lysosomal fluid (artificial lysosomal fluid, ALF). Data were expressed as mean ± SD and n = 3. (E) Energy dispersive spectrometer (EDS) analyses of elemental constituents on NCM.


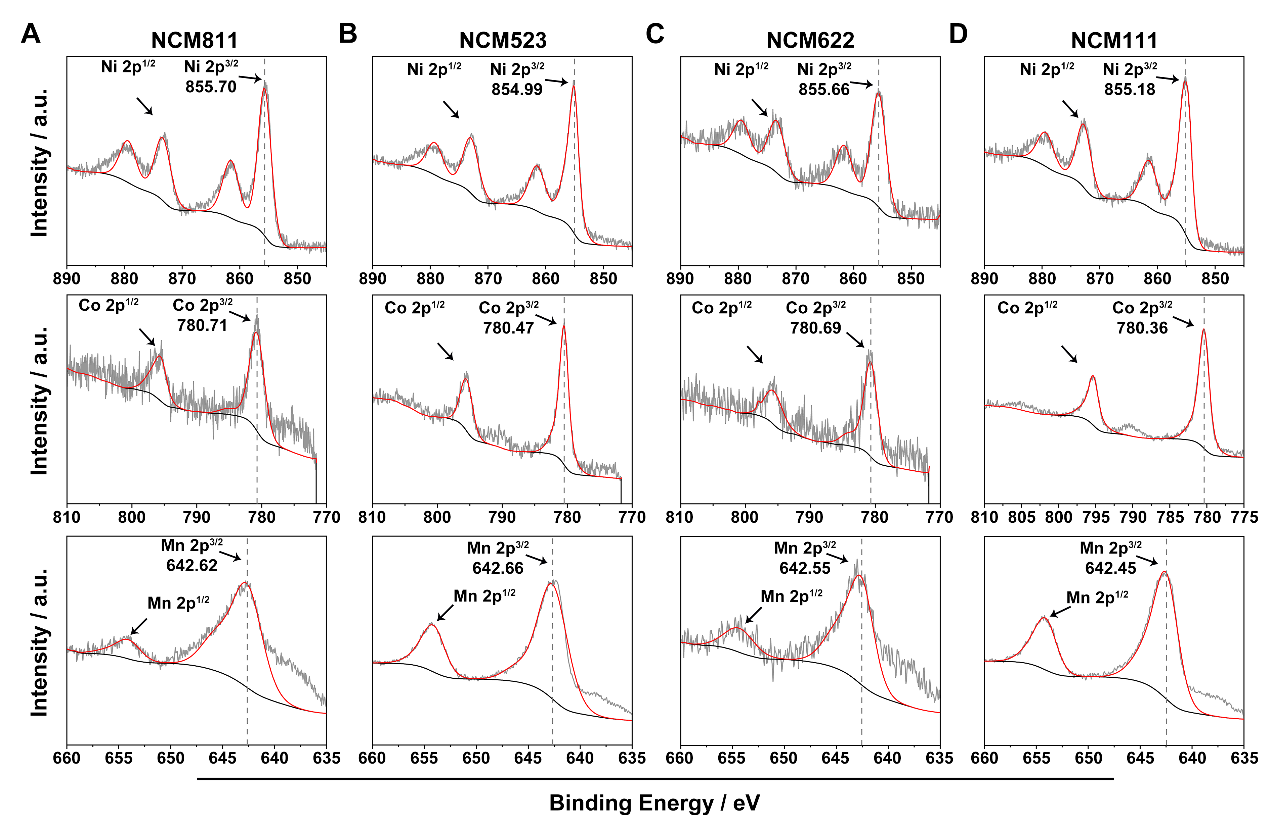


**Figure S2.** XPS spectra of NCM. Ni 2p, Co 2p, and Mn 2p for (A) NCM811, (B) NCM523, (C) NCM622, and (D) NCM111. The gray lines are raw data, the black line is the baseline, and the red line is the fitting line. XPS: X-ray photoelectron spectroscopy.


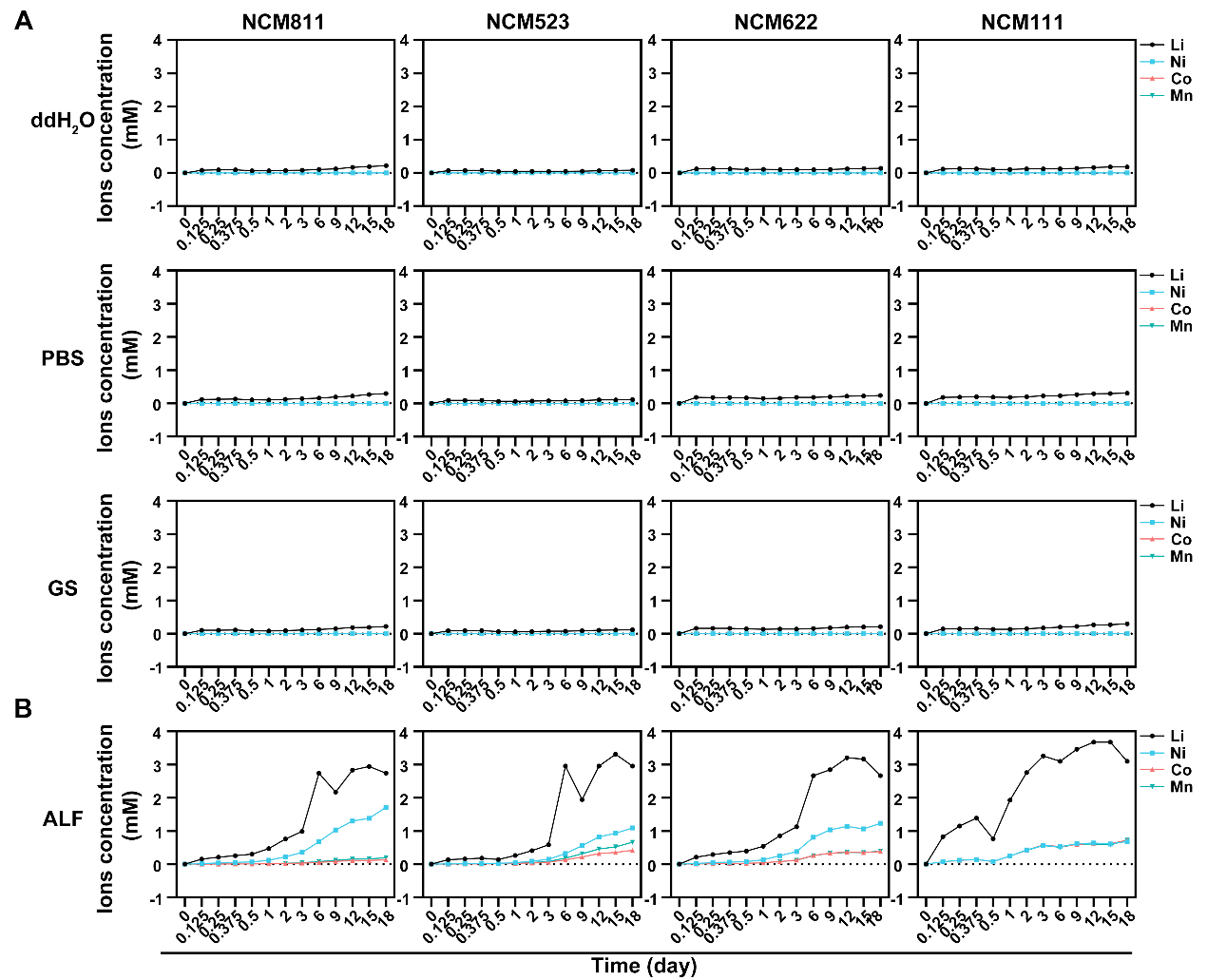


**Figure S3.** Release of elements from NCM particles over 18 days. Concentrations of released Li, Ni, Co, and Mn from NCM particles in (A) ddH_2_O, PBS, GS, and (B) ALF.


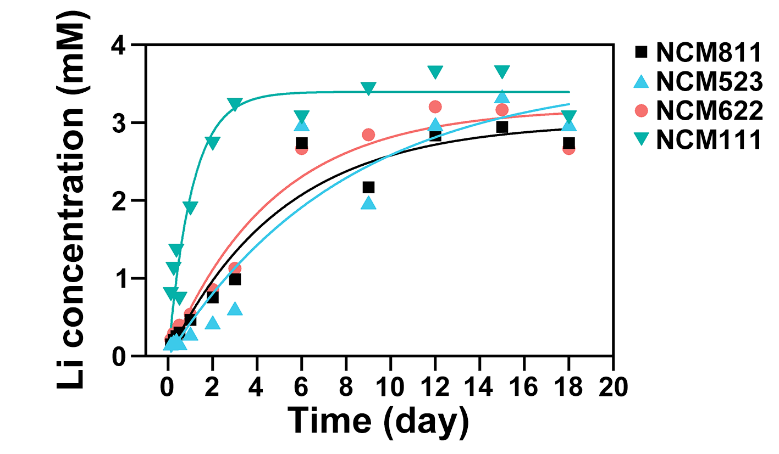


**Figure S4.** Release pattern of Li from NCM particles characterized by the first-order kinetic.

**
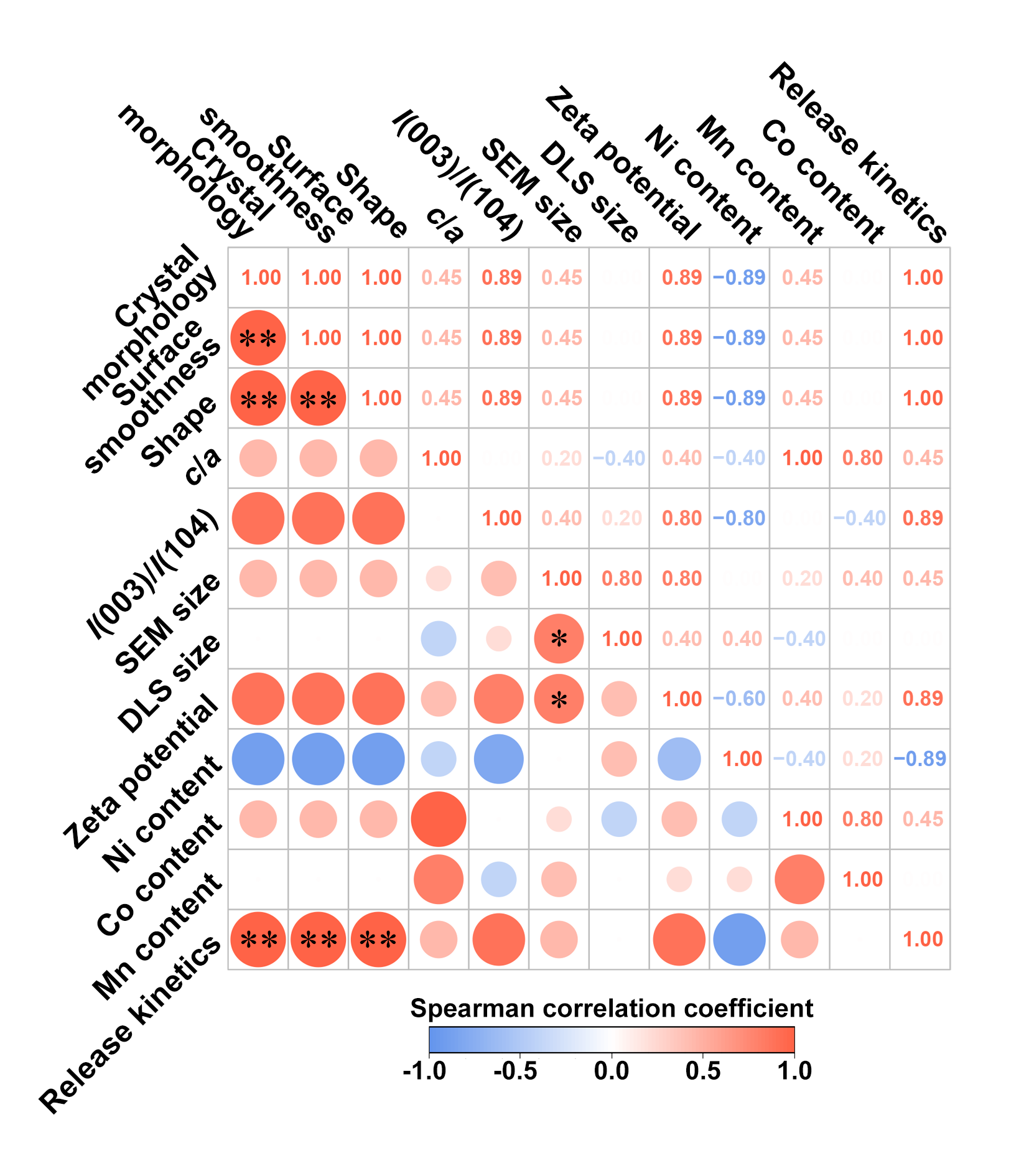
**

**Figure S5.** Correlation analysis between the element release kinetics in ALF and physicochemical properties of NCM particles. * *p* < 0.05, and ** *p* < 0.01.


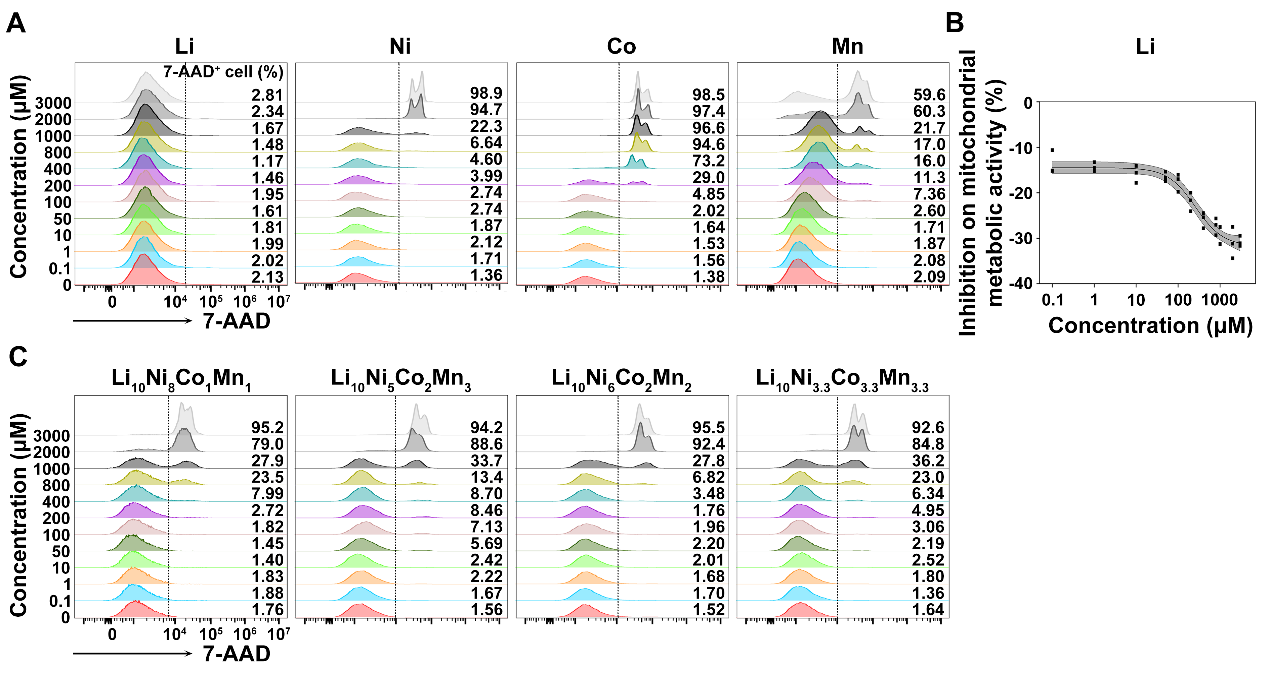


**Figure S6.** Inhibition on the cell viability and mitochondrial metabolic activity of individual element and element mixtures from NCM particles. (A) The inhibition of individual Li, Ni, Co, and Mn on cell viability. (B) The inhibition of Li on mitochondrial metabolic activity. n = 3. (C) The inhibition of element mixtures released from NCM on cell viability.


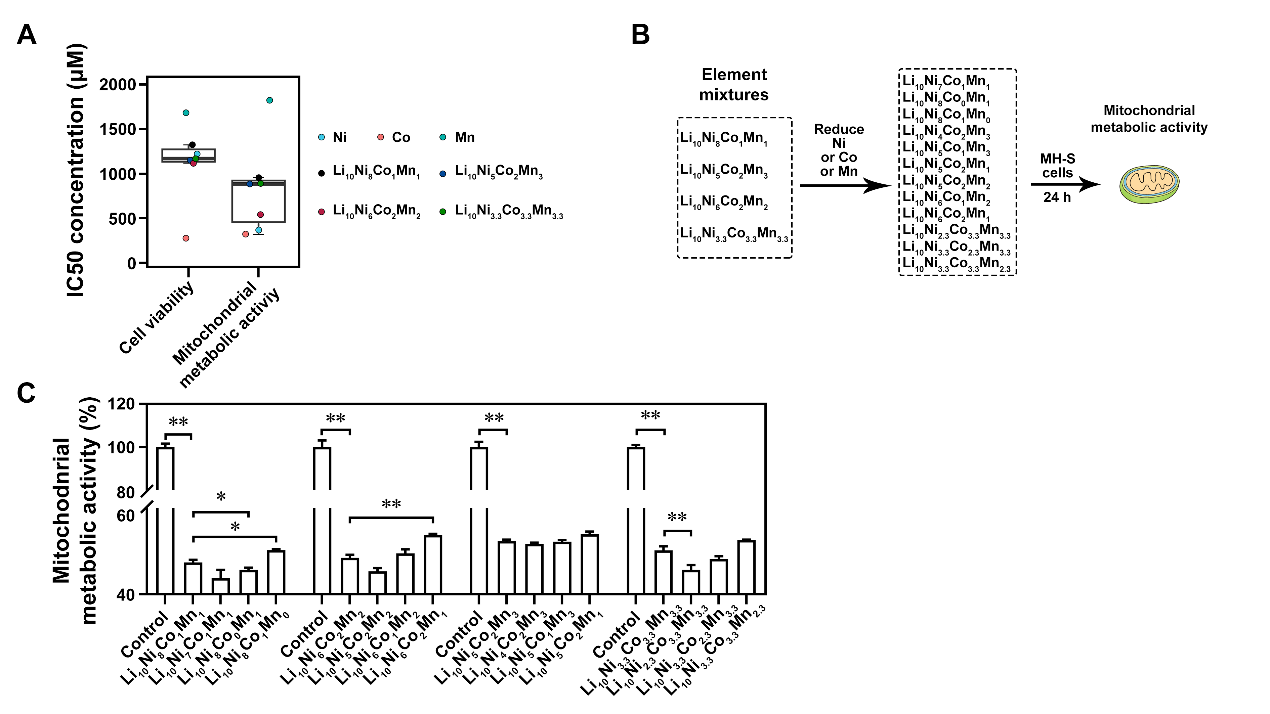


**Figure S7.** Comparison of the *IC50* concentration between cell viability and mitochondrial metabolic activity and inhibition of element mixtures on mitochondrial metabolic activity. (A) Comparison for the *IC50* concentration of cell viability and mitochondrial metabolic activity from the elements and element mixtures. (B) Schematic diagram of evaluating the interaction among Ni, Co and Mn mitochondrial metabolic activity in element mixtures from NCM particles based on the *IC50* concentration. (C) The inhibition of element mixtures from NCM with different proportion of Ni, Co, and Mn on mitochondrial metabolic activity. n = 3, * *p* < 0.05, ** *p* < 0.01. Statistical analysis was performed using one-way ANOVA followed with Dunnett's multiple comparisons test. The adjusted *p* value of control, Li_10_Ni_8_Co_0_Mn_1_ and Li_10_Ni_8_Co_1_Mn_0_ versus Li_10_Ni_8_Co_1_Mn_1_ group was 0.0008, 0.0105, and 0.0164, respectively. The adjusted *p* value of control and Li_10_Ni_6_Co_2_Mn_1_ versus Li_10_Ni_6_Co_2_Mn_2_ group was 0.0016 and 0.0068, respectively. The adjusted *p* value of control versus Li_10_Ni_5_Co_2_Mn_3_ group was 0.0022. The adjusted *p* value of control and Li_10_Ni_2.3_Co_3.3_Mn_3.3_ versus Li_10_Ni_3.3_Co_3.3_Mn_3.3_ group was 0.0006 and 0.0018, respectively. *IC50*: half maximal inhibitory concentration.


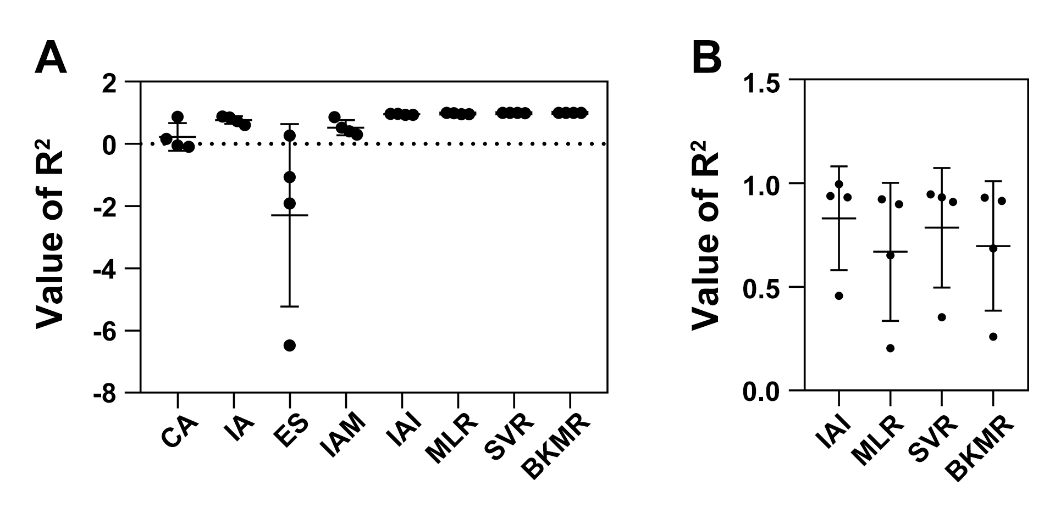


**Figure S8.** R^2^ value of toxicity from toxicity prediction model and the actual value of toxicity. (A) R^2^ value of the comparison of the toxicity from concentration addition model (CA), independent action model (IA), effect summation model (ES), integrated addition model (IAM), the IAI, multiple linear regression (MLR), support vector regression (SVR), and Bayesian kernel machine regression (BKMR) model with the toxicity of element mixtures. (B) R^2^ value of the comparison of the toxicity from MLR, SVR, and BKMR model with the actual value of toxicity based on the detected concentration of Ni, Co and Mn in their release. R^2^: R-square.


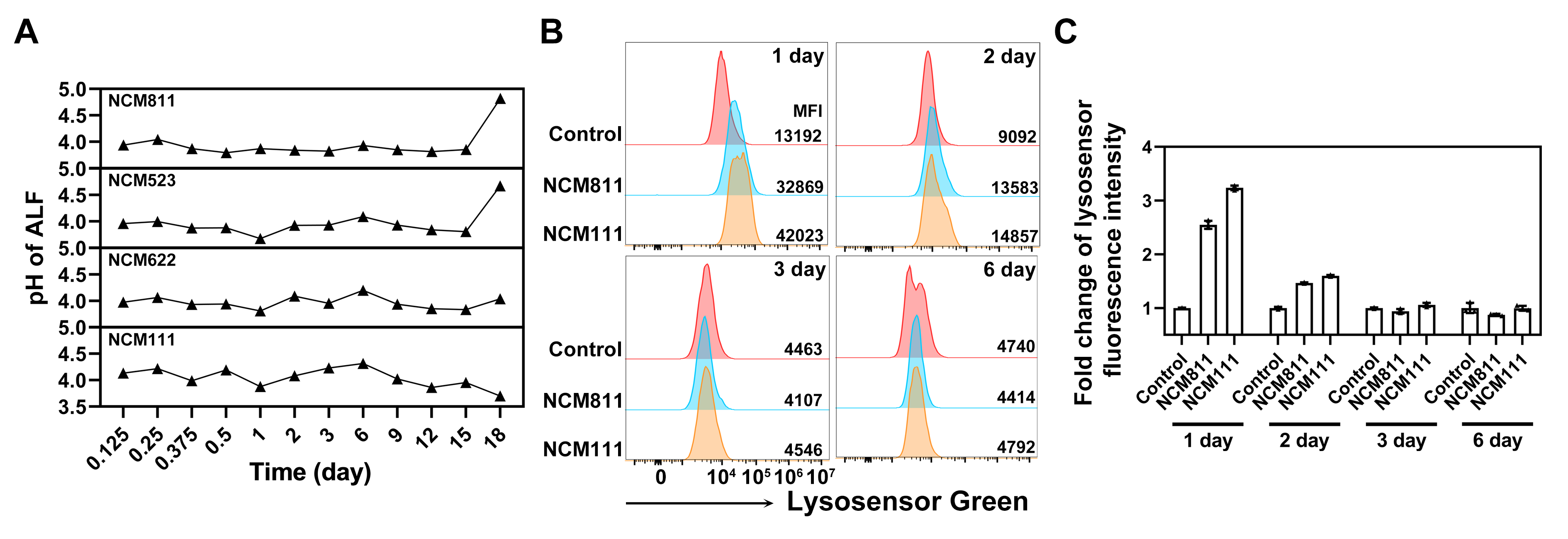


**Figure S9.** Changes of pH in alveolar lysosomal fluid and cellular lysosomes during NCM treatment. (A) The pH of GS solution during NCM particles incubation. (B) The fluorescence intensity of lysosensor green in MH-S cells treated with 200 μg/mL NCM811 or NCM111 during 3 days and (C) quantification results. n = 3.


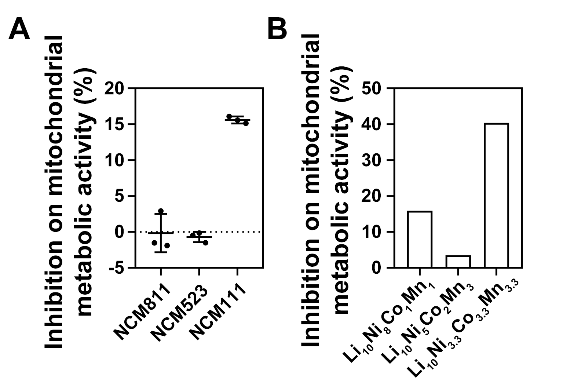


**Figure S10.** Toxicity of NCM particles and simulated released element mixtures. (A) Inhibition of 200 μg/mL NCM811, NCM523, and NCM111 on mitochondrial metabolic activity after 24 h exposure. n = 3. (B) Inhibition of simulated released element mixtures from NCM811, NCM523, and NCM111 on mitochondrial metabolic activity after 24 h exposure, the concentrations of element mixtures were based on the released elements concentration from 200 μg/mL NCM811, NCM523, and NCM111 in ALF for 24 h.


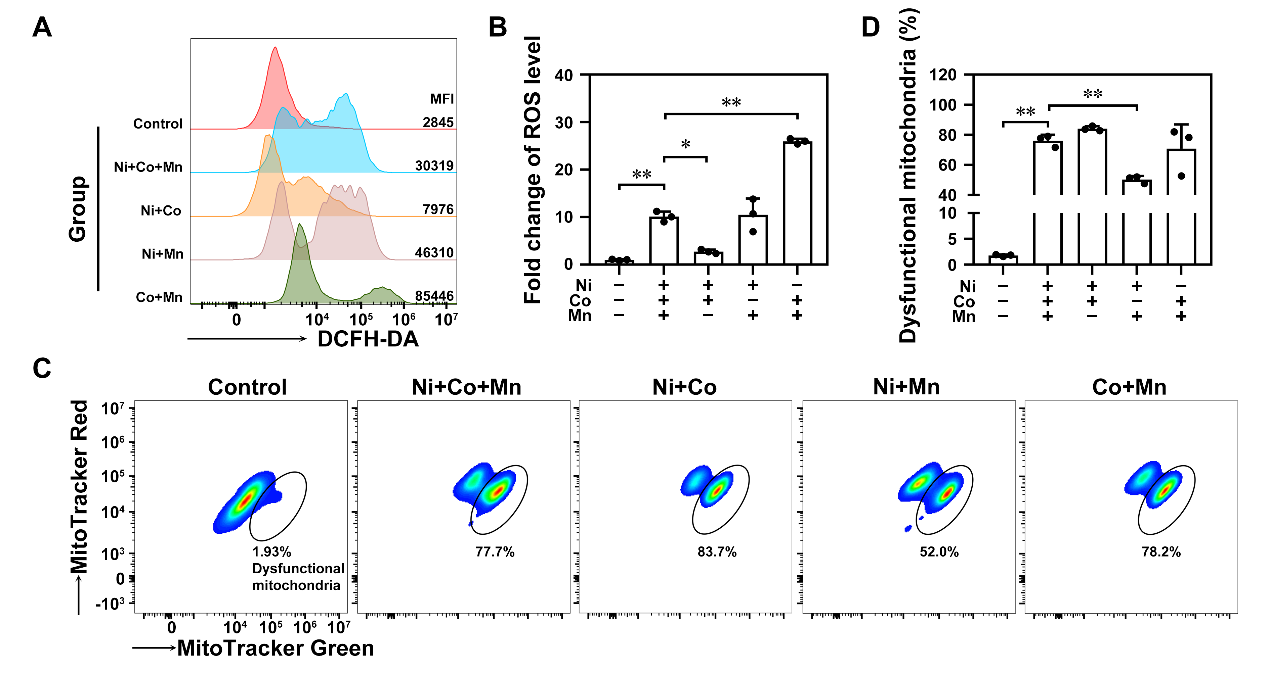


**Figure S11.** The effect of Ni, Co and Mn binary and ternary element mixtures on ROS level and dysfunctional mitochondria. (A) The effect of Ni, Co and Mn binary and ternary element mixtures on ROS level and (B) quantification results. n = 3. The adjusted *p* value of control, Ni/Co and Co/Mn versus Ni/Co/Mn group was 0.0088, 0.0108, and 0.0025, respectively. (C) The effect of Ni, Co and Mn binary and ternary element mixtures on dysfunctional mitochondria and (D) quantification results of the percent of MitoTracker Green^+^/MitoTracker Red^-^ cells. n = 3. The adjusted *p* value of control and Ni/Mn versus Ni/Co/Mn group was 0.0022 and 0.0028, respectively. * *p* < 0.05, ** *p* < 0.01. Statistical analysis was performed using one-way ANOVA followed with Dunnett's multiple comparisons test.


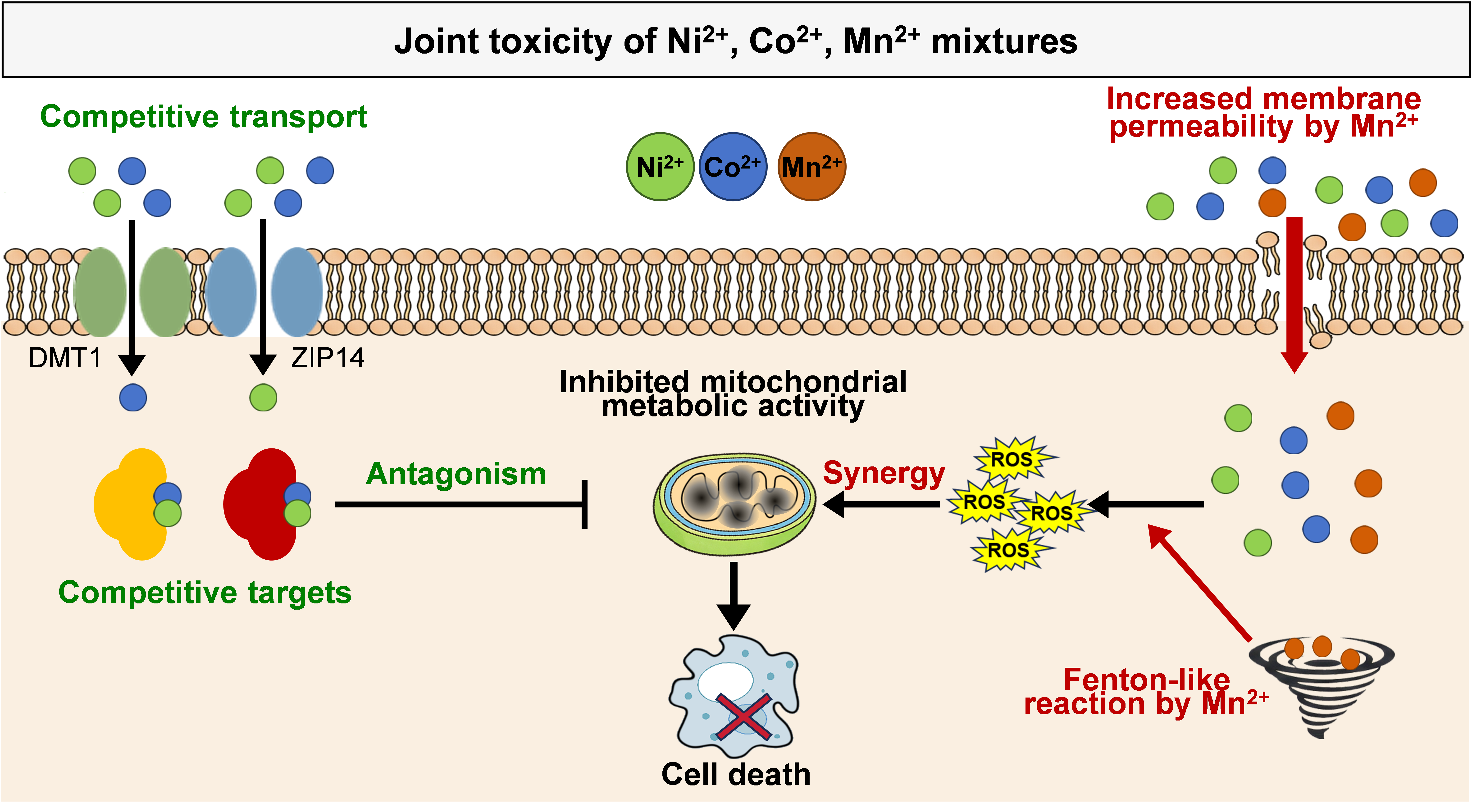


**Figure S12.** A schematic figure for the potential mechanism of Ni/Co antagonism and Mn synergy in mixture toxicity in this study.


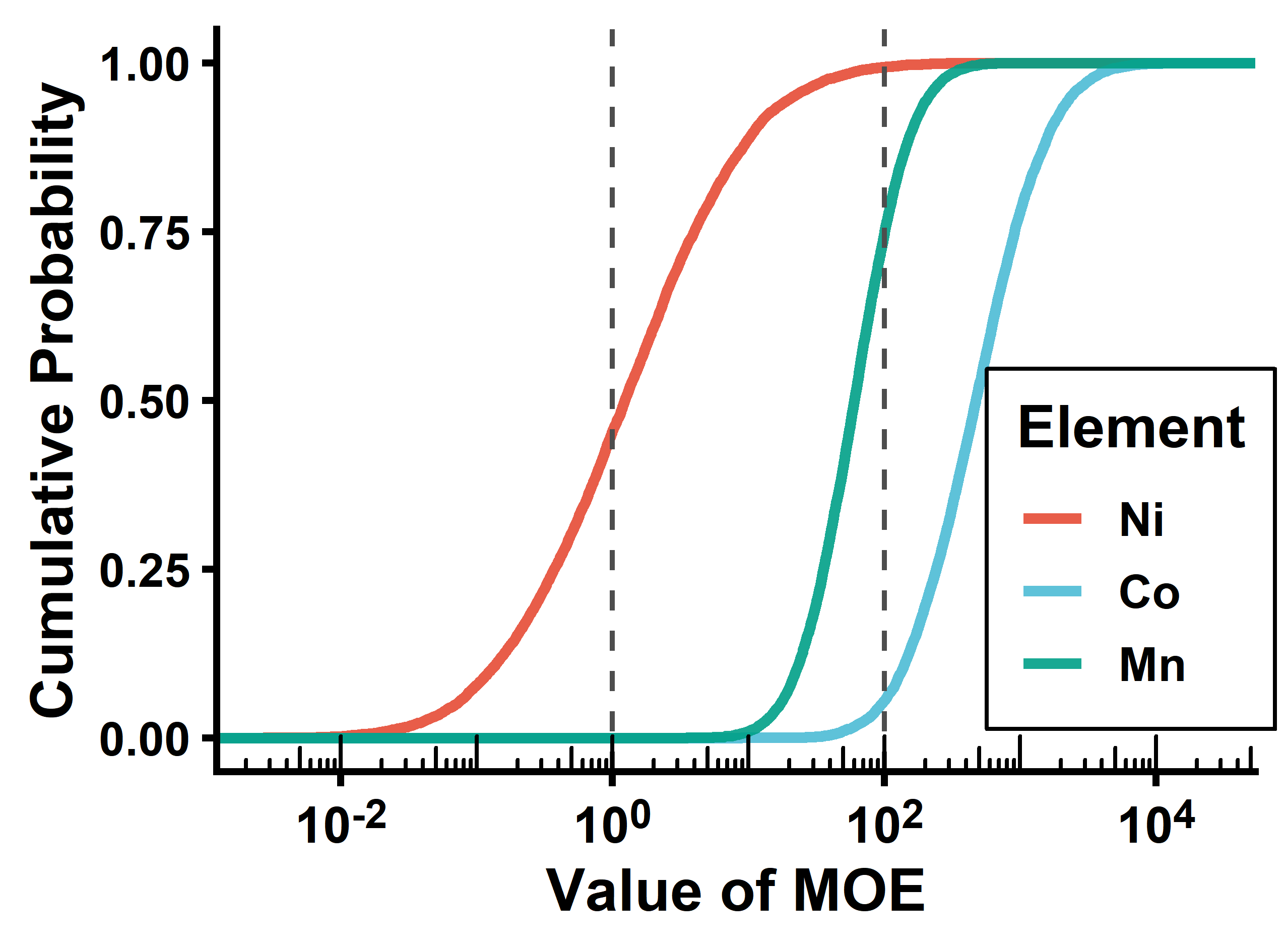


**Figure S13.** Uncertainty analysis of population risk related to NCM particle production based on individual elements. Cumulative probability of MOE was generated by Monte Carlo simulation, accounting for uncertainties in both IC_10_-based toxicity thresholds derived from mitochondrial metabolic activity assays of individual elements and population blood concentrations of Ni, Co, and Mn associated with NCM particle production. Dashed vertical lines indicate MOE values of 1 and 100.


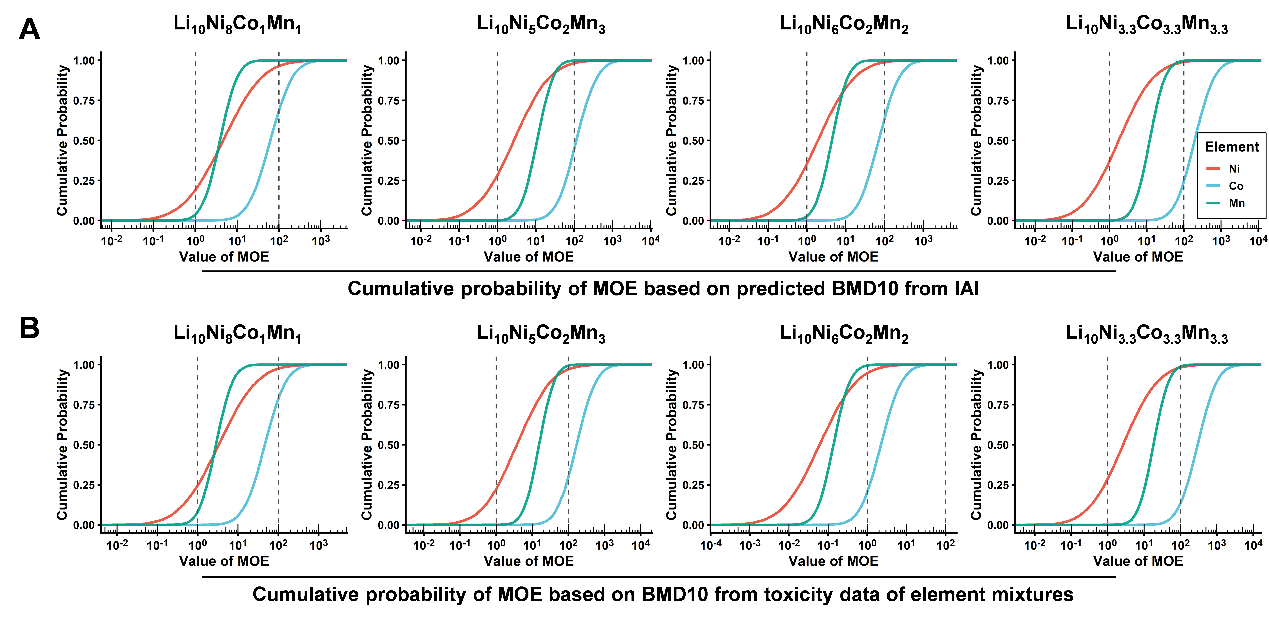


**Figure S14.** Uncertainty analysis of population risk related to NCM particle production based on NCM element mixtures. (A) Cumulative probability of MOE by Monte Carlo simulation using the IAI model. This analysis accounts for uncertainties in both IC10-based toxicity thresholds (derived from mitochondrial metabolic activity assays of NCM variants: Li₁₀Ni₈Co₁Mn₁, Li₁₀Ni₅Co₂Mn₃, Li₁₀Ni₆Co₂Mn₂, and Li₁₀Ni₃.₃Co₃.₃Mn₃.₃) and population blood concentrations of Ni, Co, and Mn. (B) Cumulative probability of MOE generated using experimental IC10 data for the same NCM mixtures. Dashed vertical lines indicate MOE values of 1 and 100.


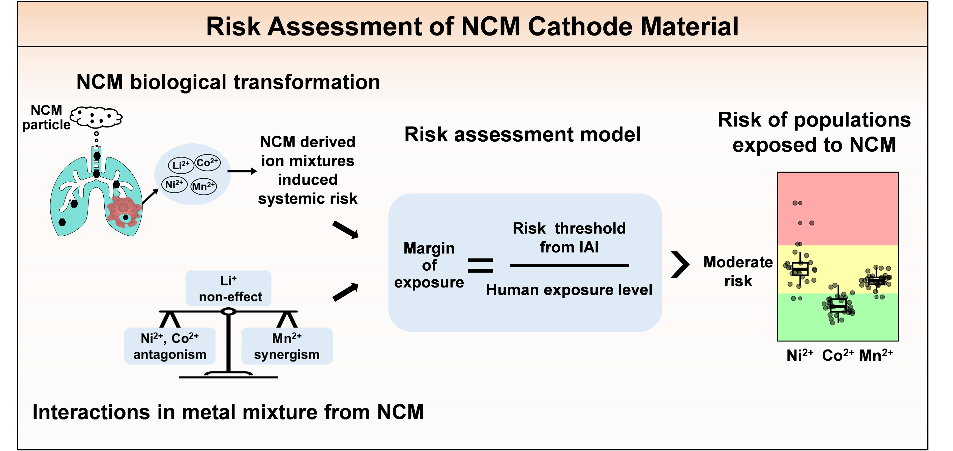


**Figure S15.** Risk assessment of NCM exposure in population with high exposure level on the basis of NCM biological transformation-derived ion mixtures and involved ion-ion interactions. Inhaled NCM particles undergo lysosomal degradation, releasing complex ion mixtures that induce systemic impact. The impact is determined by a critical balance between antagonistic Ni-Co interactions and synergistic Mn effects. To capture these complexities in risk assessment, we develop an IAI model ensuring a more accurate quantitative risk assessment. Application to NCM high-exposure populations indicates major moderate health risks.

**Table S1.** Lattice parameters of NCM particles from XRD assay.

| **NCM type** | ***a*(Å)** | ***c*(Å)** | ***c*/*a*** | **(003)** | **(104)** | ***I*(003)/*I*(104)** |
| --- | --- | --- | --- | --- | --- | --- |
| NCM811 | 2.87057 | 14.18240 | 4.941 | 3690 | 3088 | 1.195 |
| NCM523 | 2.86752 | 14.22530 | 4.961 | 9862 | 8914 | 1.106 |
| NCM622 | 2.86890 | 14.21660 | 4.955 | 21215 | 13309 | 1.594 |
| NCM111 | 2.85402 | 14.20289 | 4.976 | 8194 | 6171 | 1.328 |

XRD: X-ray diffraction.

**Table S2.** Calculated concentration for complete dissolution and detected concentrations of released Li, Ni, Co, and Mn from NCM in the ALF after 18 days of incubation.

| **NCM type** | **Element** | **Particle concentration (μg/mL)** | **Calculated concentration (mM)** | **Detected concentration (mM)** |
| --- | --- | --- | --- | --- |
| NCM811 | Li | 200 | 2.06 | 2.74 |
|  | Ni | 200 | 1.64 | 1.71 |
|  | Co | 200 | 0.21 | 0.13 |
|  | Mn | 200 | 0.21 | 0.19 |
| NCM523 | Li | 200 | 2.07 | 2.95 |
|  | Ni | 200 | 1.04 | 1.09 |
|  | Co | 200 | 0.41 | 0.42 |
|  | Mn | 200 | 0.62 | 0.66 |
| NCM622 | Li | 200 | 2.06 | 2.67 |
|  | Ni | 200 | 1.24 | 1.23 |
|  | Co | 200 | 0.41 | 0.38 |
|  | Mn | 200 | 0.41 | 0.39 |
| NCM111 | Li | 200 | 2.07 | 3.10 |
|  | Ni | 200 | 0.69 | 0.68 |
|  | Co | 200 | 0.69 | 0.72 |
|  | Mn | 200 | 0.69 | 0.72 |

**Table S3.** Value of *IC50* for individual elements and element mixtures on mitochondrial metabolic activity.

| **Elements or element mixtures** | ***IC50* (μM ± SE)** | **Concentration of Ni in *IC50*** | **Concentration of Co in *IC50*** | **Concentration of Mn in *IC50*** |
| --- | --- | --- | --- | --- |
| Ni | 372.3090 ± 26.5056 | 372.3090 | - | - |
| Co | 321.7393 ± 20.2757 | - | 321.7393 | - |
| Mn | 1828.5483 ± 104.5022 | - | - | 1828.5483 |
| Li_10_Ni_8_Co_1_Mn_1_ | 957.2196 ± 42.2700 | 765.77568 | 95.72196 | 95.72196 |
| Li_10_Ni_5_Co_2_Mn_3_ | 887.2415 ± 29.0421 | 443.620745 | 177.4483 | 266.172447 |
| Li_10_Ni_6_Co_2_Mn_2_ | 543.1419 ± 42.7404 | 325.885152 | 108.6284 | 108.628384 |
| Li_10_Ni_3.3_Co_3.3_Mn_3.3_ | 893.7998 ± 54.8289 | 297.933253 | 297.9333 | 297.933253 |

Data were shown mean ± standard error (SE).

**Table S4.** Calculation of AI, MTI, and TU for evaluating the interactions of individual elements in the mixtures.

| **Element mixtures** | **Inhibition rate (%)** | **C_Ni_ in mixtures (μM)** | **C_Co_ in mixtures (μM)** | **C_Mn_ in mixtures (μM)** | **TU_Ni_** | **TU_Co_** | **TU_Mn_** | **M** | **M0** | **Interaction of TU** | **AI** | **Interaction of AI** | **MTI** | **Interaction of MTI** |
| --- | --- | --- | --- | --- | --- | --- | --- | --- | --- | --- | --- | --- | --- | --- |
| Li_10_Ni_8_Co_1_Mn_1_ | 5 | 20.45 | 2.56 | 2.56 | 0.94 | 0.02 | 0.01 | 0.97 | 1.03 | synergistic | 0.03 | synergistic | 1.87 | synergistic |
|  | 10 | 116.89 | 14.61 | 14.61 | 2.72 | 0.10 | 0.05 | 2.87 | 1.05 | antagonistic | -1.87 | antagonistic | -19.42 | antagonistic |
|  | 15 | 194.36 | 24.29 | 24.29 | 2.94 | 0.15 | 0.06 | 3.15 | 1.07 | antagonistic | -2.15 | antagonistic | -15.99 | antagonistic |
|  | 20 | 268.17 | 33.52 | 33.52 | 2.92 | 0.18 | 0.06 | 3.17 | 1.08 | antagonistic | -2.17 | antagonistic | -13.17 | antagonistic |
|  | 25 | 341.91 | 42.74 | 42.74 | 2.82 | 0.21 | 0.06 | 3.10 | 1.10 | antagonistic | -2.10 | antagonistic | -11.02 | antagonistic |
|  | 30 | 417.51 | 52.19 | 52.19 | 2.70 | 0.24 | 0.06 | 3.00 | 1.11 | antagonistic | -2.00 | antagonistic | -9.32 | antagonistic |
|  | 35 | 496.37 | 62.05 | 62.05 | 2.55 | 0.26 | 0.06 | 2.87 | 1.13 | antagonistic | -1.87 | antagonistic | -7.90 | antagonistic |
|  | 40 | 579.78 | 72.47 | 72.47 | 2.40 | 0.28 | 0.06 | 2.73 | 1.14 | antagonistic | -1.73 | antagonistic | -6.68 | antagonistic |
|  | 45 | 669.09 | 83.64 | 83.64 | 2.23 | 0.29 | 0.06 | 2.58 | 1.15 | antagonistic | -1.58 | antagonistic | -5.59 | antagonistic |
|  | 50 | 765.78 | 95.72 | 95.72 | 2.06 | 0.30 | 0.05 | 2.41 | 1.17 | antagonistic | -1.41 | antagonistic | -4.59 | antagonistic |
|  | 55 | 871.65 | 108.96 | 108.96 | 1.87 | 0.30 | 0.05 | 2.22 | 1.19 | antagonistic | -1.22 | antagonistic | -3.64 | antagonistic |
|  | 60 | 988.93 | 123.62 | 123.62 | 1.66 | 0.30 | 0.04 | 2.00 | 1.21 | antagonistic | -1.00 | antagonistic | -2.70 | antagonistic |
|  | 65 | 1120.50 | 140.06 | 140.06 | 1.43 | 0.28 | 0.04 | 1.75 | 1.23 | antagonistic | -0.75 | antagonistic | -1.75 | antagonistic |
|  | 70 | 1270.16 | 158.77 | 158.77 | 1.17 | 0.25 | 0.04 | 1.46 | 1.25 | antagonistic | -0.46 | antagonistic | -0.72 | antagonistic |
|  | 75 | 1443.14 | 180.39 | 180.39 | 0.87 | 0.19 | 0.03 | 1.09 | 1.25 | partly additive | -0.09 | antagonistic | 0.61 | partly additive |
| Li_10_Ni_5_Co_2_Mn_3_ | 5 | 95.39 | 38.16 | 57.23 | 4.40 | 0.28 | 0.26 | 4.93 | 1.12 | antagonistic | -3.93 | antagonistic | -12.83 | antagonistic |
|  | 10 | 132.73 | 53.09 | 79.64 | 2.22 | 0.25 | 0.18 | 2.66 | 1.20 | antagonistic | -1.66 | antagonistic | -4.44 | antagonistic |
|  | 15 | 170.57 | 68.23 | 102.34 | 2.01 | 0.32 | 0.19 | 2.52 | 1.26 | antagonistic | -1.52 | antagonistic | -3.07 | antagonistic |
|  | 20 | 208.79 | 83.52 | 125.27 | 1.86 | 0.37 | 0.19 | 2.42 | 1.31 | antagonistic | -1.42 | antagonistic | -2.32 | antagonistic |
|  | 25 | 247.33 | 98.93 | 148.40 | 1.72 | 0.42 | 0.19 | 2.33 | 1.35 | antagonistic | -1.33 | antagonistic | -1.80 | antagonistic |
|  | 30 | 286.15 | 114.46 | 171.69 | 1.60 | 0.45 | 0.18 | 2.23 | 1.40 | antagonistic | -1.23 | antagonistic | -1.40 | antagonistic |
|  | 35 | 325.22 | 130.09 | 195.13 | 1.47 | 0.48 | 0.17 | 2.12 | 1.44 | antagonistic | -1.12 | antagonistic | -1.06 | antagonistic |
|  | 40 | 364.49 | 145.80 | 218.69 | 1.35 | 0.50 | 0.16 | 2.00 | 1.49 | antagonistic | -1.00 | antagonistic | -0.75 | antagonistic |
|  | 45 | 403.96 | 161.59 | 242.38 | 1.22 | 0.50 | 0.15 | 1.87 | 1.53 | antagonistic | -0.87 | antagonistic | -0.46 | antagonistic |
|  | 50 | 443.62 | 177.45 | 266.17 | 1.09 | 0.50 | 0.13 | 1.72 | 1.59 | antagonistic | -0.72 | antagonistic | -0.18 | antagonistic |
|  | 55 | 483.47 | 193.39 | 290.08 | 0.95 | 0.49 | 0.12 | 1.56 | 1.64 | partly additive | -0.56 | antagonistic | 0.10 | partly additive |
|  | 60 | 523.57 | 209.43 | 314.14 | 0.81 | 0.47 | 0.10 | 1.38 | 1.70 | partly additive | -0.38 | antagonistic | 0.39 | partly additive |
|  | 65 | 564.11 | 225.64 | 338.47 | 0.67 | 0.42 | 0.09 | 1.18 | 1.77 | partly additive | -0.18 | antagonistic | 0.71 | partly additive |
|  | 70 | 605.60 | 242.24 | 363.36 | 0.52 | 0.36 | 0.08 | 0.96 | 1.84 | synergistic | 0.04 | synergistic | 1.07 | synergistic |
|  | 75 | 649.43 | 259.77 | 389.66 | 0.37 | 0.25 | 0.06 | 0.68 | 1.84 | synergistic | 0.32 | synergistic | 1.64 | synergistic |
| Li_10_Ni_6_Co_2_Mn_2_ | 10 | 2.13 | 0.71 | 0.71 | 0.05 | 0.00 | 0.00 | 0.06 | 1.14 | synergistic | 0.94 | synergistic | 22.76 | synergistic |
|  | 15 | 13.28 | 4.43 | 4.43 | 0.20 | 0.03 | 0.01 | 0.24 | 1.19 | synergistic | 0.76 | synergistic | 9.40 | synergistic |
|  | 20 | 33.30 | 11.10 | 11.10 | 0.36 | 0.06 | 0.02 | 0.44 | 1.23 | synergistic | 0.56 | synergistic | 4.98 | synergistic |
|  | 25 | 61.82 | 20.61 | 20.61 | 0.51 | 0.10 | 0.03 | 0.64 | 1.26 | synergistic | 0.36 | synergistic | 2.88 | synergistic |
|  | 30 | 98.64 | 32.88 | 32.88 | 0.64 | 0.15 | 0.04 | 0.83 | 1.30 | synergistic | 0.17 | synergistic | 1.72 | synergistic |
|  | 35 | 143.57 | 47.86 | 47.86 | 0.74 | 0.20 | 0.05 | 0.99 | 1.34 | synergistic | 0.01 | synergistic | 1.05 | synergistic |
|  | 40 | 196.49 | 65.50 | 65.50 | 0.81 | 0.25 | 0.05 | 1.12 | 1.37 | partly additive | -0.12 | antagonistic | 0.65 | partly additive |
|  | 45 | 257.29 | 85.76 | 85.76 | 0.86 | 0.30 | 0.06 | 1.21 | 1.41 | partly additive | -0.21 | antagonistic | 0.44 | partly additive |
|  | 50 | 325.89 | 108.63 | 108.63 | 0.88 | 0.34 | 0.06 | 1.27 | 1.45 | partly additive | -0.27 | antagonistic | 0.36 | partly additive |
|  | 55 | 402.19 | 134.06 | 134.06 | 0.86 | 0.37 | 0.06 | 1.29 | 1.50 | partly additive | -0.29 | antagonistic | 0.37 | partly additive |
|  | 60 | 486.15 | 162.05 | 162.05 | 0.82 | 0.39 | 0.06 | 1.26 | 1.55 | partly additive | -0.26 | antagonistic | 0.47 | partly additive |
|  | 65 | 577.68 | 192.56 | 192.56 | 0.74 | 0.39 | 0.05 | 1.18 | 1.60 | partly additive | -0.18 | antagonistic | 0.65 | partly additive |
|  | 70 | 676.74 | 225.58 | 225.58 | 0.62 | 0.36 | 0.05 | 1.03 | 1.66 | partly additive | -0.03 | antagonistic | 0.93 | partly additive |
|  | 75 | 783.28 | 261.09 | 261.09 | 0.47 | 0.27 | 0.04 | 0.79 | 1.66 | synergistic | 0.21 | synergistic | 1.47 | synergistic |
| Li_10_Ni_3.3_Co_3.3_Mn_3.3_ | 5 | 59.68 | 59.68 | 59.68 | 2.75 | 0.44 | 0.27 | 3.46 | 1.26 | antagonistic | -2.46 | antagonistic | -4.42 | antagonistic |
|  | 10 | 93.40 | 93.40 | 93.40 | 2.17 | 0.62 | 0.30 | 3.09 | 1.42 | antagonistic | -2.09 | antagonistic | -2.20 | antagonistic |
|  | 15 | 120.55 | 120.55 | 120.55 | 1.82 | 0.73 | 0.29 | 2.84 | 1.56 | antagonistic | -1.84 | antagonistic | -1.35 | antagonistic |
|  | 20 | 145.24 | 145.24 | 145.24 | 1.58 | 0.80 | 0.28 | 2.65 | 1.68 | antagonistic | -1.65 | antagonistic | -0.88 | antagonistic |
|  | 25 | 169.00 | 169.00 | 169.00 | 1.40 | 0.85 | 0.26 | 2.50 | 1.79 | antagonistic | -1.50 | antagonistic | -0.57 | antagonistic |
|  | 30 | 192.65 | 192.65 | 192.65 | 1.24 | 0.88 | 0.23 | 2.36 | 1.90 | antagonistic | -1.36 | antagonistic | -0.34 | antagonistic |
|  | 35 | 216.82 | 216.82 | 216.82 | 1.12 | 0.91 | 0.22 | 2.24 | 2.01 | antagonistic | -1.24 | antagonistic | -0.16 | antagonistic |
|  | 40 | 242.04 | 242.04 | 242.04 | 1.00 | 0.92 | 0.20 | 2.12 | 2.12 | antagonistic | -1.12 | antagonistic | 0.00 | antagonistic |
|  | 45 | 268.86 | 268.86 | 268.86 | 0.90 | 0.93 | 0.18 | 2.01 | 2.16 | partly additive | -1.01 | antagonistic | 0.10 | partly additive |
|  | 50 | 297.93 | 297.93 | 297.93 | 0.80 | 0.93 | 0.16 | 1.89 | 2.04 | partly additive | -0.89 | antagonistic | 0.11 | partly additive |
|  | 55 | 330.06 | 330.06 | 330.06 | 0.71 | 0.91 | 0.15 | 1.77 | 1.94 | partly additive | -0.77 | antagonistic | 0.14 | partly additive |
|  | 60 | 366.35 | 366.35 | 366.35 | 0.61 | 0.88 | 0.13 | 1.63 | 1.85 | partly additive | -0.63 | antagonistic | 0.21 | partly additive |
|  | 65 | 408.37 | 408.37 | 408.37 | 0.52 | 0.83 | 0.12 | 1.47 | 1.77 | partly additive | -0.47 | antagonistic | 0.33 | partly additive |
|  | 70 | 458.53 | 458.53 | 458.53 | 0.42 | 0.73 | 0.10 | 1.26 | 1.72 | partly additive | -0.26 | antagonistic | 0.58 | partly additive |
|  | 75 | 520.81 | 520.81 | 520.81 | 0.32 | 0.54 | 0.09 | 0.94 | 1.75 | synergistic | 0.06 | synergistic | 1.11 | synergistic |

AI: additive index, MTI: mixture toxicity index, TU: toxic unit.

**Table S5.** The RMSE and R^2^ of model-calculated toxicity and the actual toxicity of the element mixture.

| **Element mixture** | **Toxicity prediction model** | **RMSE** | **R^2^** |
| --- | --- | --- | --- |
| Li_10_Ni_8_Co_1_Mn_1_ | CA | 19.2570 | -0.0447 |
|  | IA | 9.3925 | 0.8824 |
|  | ES | 23.3736 | 0.2716 |
|  | IAM | 24.0464 | 0.3044 |
|  | IAI | 5.1389 | 0.9682 |
|  | LR | 6.7010 | 0.9561 |
|  | SVR | 2.8622 | 0.9920 |
|  | BKMR | 0.6686 | 0.9996 |
| Li_10_Ni_5_Co_2_Mn_3_ | CA | 6.4490 | 0.8685 |
|  | IA | 9.5960 | 0.7369 |
|  | ES | 17.0706 | -1.9141 |
|  | IAM | 9.7163 | 0.8597 |
|  | IAI | 6.4875 | 0.9375 |
|  | LR | 7.1550 | 0.9579 |
|  | SVR | 2.6956 | 0.9940 |
|  | BKMR | 0.7215 | 0.9996 |
| Li_10_Ni_6_Co_2_Mn_2_ | CA | 23.2913 | 0.1625 |
|  | IA | 8.3647 | 0.8501 |
|  | ES | 20.6245 | -1.0624 |
|  | IAM | 16.8693 | 0.5257 |
|  | IAI | 6.0007 | 0.9400 |
|  | LR | 4.1902 | 0.9837 |
|  | SVR | 2.4794 | 0.9943 |
|  | BKMR | 0.9609 | 0.9991 |
| Li_10_Ni_3.3_Co_3.3_Mn_3.3_ | CA | 22.1075 | -0.0889 |
|  | IA | 17.0940 | 0.6104 |
|  | ES | 31.3339 | -6.4805 |
|  | IAM | 22.2422 | 0.4049 |
|  | IAI | 5.5353 | 0.9631 |
|  | LR | 1.7445 | 0.9972 |
|  | SVR | 2.5381 | 0.9941 |
|  | BKMR | 0.7069 | 0.9995 |

Concentration addition model (CA), independent action model (IA), effect summation model (ES), integrated addition model (IAM), the integrated addition and interaction model (IAI), linear regression (LR), support vector regression (SVR), and Bayesian kernel machine regression (BKMR). RMSE: root-mean-square error.

**Table S6.** Levels of Li, Ni, Co and Mn in the blood of occupational populations exposed to NCM particles.

| **Ions** | **Minimum concentration (nM)** | ***P5* concentration (nM)** | **Median concentration (nM)** | ***P95* concentration (nM)** | **Maximum concentration (nM)** |
| --- | --- | --- | --- | --- | --- |
| Li | 94.380 | 179.395 | 740.490 | 1942.363 | 2296.398 |
| Ni | 0 | 0 | 255.512 | 29643.12 | 200595.553 |
| Co | 4.361 | 5.701 | 20.786 | 132.608 | 157.840 |
| Mn | 76.559 | 128.254 | 372.420 | 1203.994 | 1306.236 |

**Table S7.** Release parameter for Ni, Co, and Mn released from NCM particles.

| **NCM type** | **Parameter** | **Ni** | **Co** | **Mn** |
| --- | --- | --- | --- | --- |
| NCM811 | *k* | 0.8405 | 0.0645 | 0.0950 |
| NCM523 | *k* | 0.5109 | 0.1940 | 0.2951 |
| NCM622 | C_max_ | 0.6103 | 0.1924 | 0.1973 |
| NCM111 | C_max_ | 0.3335 | 0.3374 | 0.3291 |

Relative proportion of *k* for Ni, Co, and Mn in zero-order kinetic of NCM811 and NCM523, and relative proportion of C_max_ for Ni, Co, and Mn in first-order kinetic of NCM622 and NCM111.

**Table S8.** Uncertainty analysis of population risk related to NCM particle production.

|  | **Element** | **Median of MOE** | **CI2.5 of MOE** | **CI97.5 of MOE** | **Probability of MOE＜1 (%)** | **Probability of MOE＜100 (%)** |
| --- | --- | --- | --- | --- | --- | --- |
| Individual elements | Ni | 1.2324 | 0.0412 | 1.2324 | 45.4800 | 99.3800 |
|  | Co | 472.6429 | 70.9736 | 472.6429 | 0.0000 | 5.5200 |
|  | Mn | 60.2001 | 13.3617 | 60.2001 | 0.0000 | 74.6300 |
| NCM811 | Ni | 3.3310 | 0.1087 | 100.6298 | 24.6000 | 97.4600 |
|  | Co | 46.0153 | 6.8508 | 315.2336 | 0.0000 | 78.5400 |
|  | Mn | 2.8243 | 0.6545 | 13.0935 | 8.2700 | 100.0000 |
| NCM523 | Ni | 3.7665 | 0.1224 | 110.4951 | 22.1800 | 97.1300 |
|  | Co | 168.4747 | 25.0111 | 1144.5845 | 0.0000 | 30.0100 |
|  | Mn | 15.3132 | 3.4973 | 68.8389 | 0.0300 | 99.3600 |
| NCM622 | Ni | 0.0605 | 0.0020 | 1.8479 | 94.5900 | 100.0000 |
|  | Co | 2.2263 | 0.3314 | 15.4039 | 20.6500 | 100.0000 |
|  | Mn | 0.1376 | 0.0306 | 0.6376 | 99.4600 | 100.0000 |
| NCM111 | Ni | 2.6493 | 0.0893 | 77.1388 | 28.7100 | 98.2500 |
|  | Co | 295.7061 | 43.8993 | 1951.6683 | 0.0000 | 13.4300 |
|  | Mn | 18.1221 | 4.1830 | 80.8706 | 0.0100 | 98.8400 |
| NCM811-IAI | Ni | 4.6385 | 0.1476 | 132.8551 | 18.9400 | 96.3400 |
|  | Co | 62.1729 | 9.4139 | 426.3969 | 0.0000 | 68.5100 |
|  | Mn | 3.8771 | 0.8733 | 17.3302 | 3.6900 | 100.0000 |
| NCM523- IAI | Ni | 2.6545 | 0.0888 | 79.1577 | 28.3700 | 98.1800 |
|  | Co | 113.4659 | 17.5894 | 782.7783 | 0.0000 | 44.7200 |
|  | Mn | 10.7537 | 2.2963 | 47.6221 | 0.0800 | 99.8400 |
| NCM622- IAI | Ni | 1.9153 | 0.0650 | 58.4796 | 35.4300 | 98.7700 |
|  | Co | 70.6231 | 10.9114 | 476.0108 | 0.0000 | 63.6800 |
|  | Mn | 4.3959 | 0.9731 | 19.4863 | 2.7600 | 99.9900 |
| NCM111- IAI | Ni | 1.7589 | 0.0580 | 54.5941 | 36.9900 | 99.0700 |
|  | Co | 192.9638 | 29.4893 | 1305.1264 | 0.0000 | 24.5700 |
|  | Mn | 12.0238 | 2.7263 | 55.3822 | 0.0700 | 99.6900 |

**Table S9.** Components and relevant concentrations in GS.

| **Components** | **Concentration (mg/L)** |
| --- | --- |
| MgCl_2_ | 96 |
| NaCl | 6020 |
| KCl | 298 |
| Na_2_HPO_4_ | 126 |
| Na_2_SO_4_ | 63 |
| CaCl_2_·2H_2_O | 368 |
| C_2_H_3_O_2_Na | 574 |
| NaHCO_3_ | 2600 |
| C_6_H_5_Na_3_O_7_·2H_2_O | 97 |

**Table S10.** Components and relevant concentrations in ALF.

| **Components** | **Concentration (mg/L)** |
| --- | --- |
| MgCl_2_ | 50 |
| NaCl | 3210 |
| Na_2_HPO_4_ | 71 |
| Na_2_SO_4_ | 39 |
| CaCl_2_·2H_2_O | 128 |
| HOC(COONa)(CH_2_COONa)_2_ | 77 |
| NaOH | 6000 |
| citric acid | 20800 |
| glycine | 59 |
| Na_2_C_4_H_4_O_6_·2H_2_O | 90 |
| NaC_3_H_5_O_3_ | 85 |
| NaOCOCOCH_3_ | 86 |

pH of ALF = 4.5 ± 1

**Table S11.** Parameters and measurement conditions of ICP-OES.

| **Parameter and accessories** |  |
| --- | --- |
| Nebulizer | Meinhard C1 |
| Spray chamber | Glass swirl atomizing chamber |
| Carrier gas | Argon |
| Plasma gas flow rate (L/min) | 15 |
| Auxiliary gas flow rate (L/min) | 0.2 |
| Nebulization gas flow rate (L/min) | 0.8 |
| Plasma viewing | Axial |
| Generator power (W) | 1300 |
| Li wavelengths | 670.784 |
| Ni wavelengths | 231.604 |
| Co wavelengths | 228.620 |
| Mn wavelengths | 257.610 |

**Table S12.** Experimental design for the mixture system of Li, Ni, Co, and Mn.

| **Group** | **Proportion of Li/Ni/Co/Mn** | **LiCl (μM)** | **NiCl_2_·6H_2_O (μM)** | **CoCl_2_·6H_2_O (μM)** | **MnCl_2_·4H_2_O (μM)** |
| --- | --- | --- | --- | --- | --- |
| Control | 0/0/0/0 | 0 | 0 | 0 | 0 |
| NCM811 | 10/8/1/1 | 0.1 | 0.08 | 0.01 | 0.01 |
|  |  | 1 | 0.8 | 0.1 | 0.1 |
|  |  | 10 | 8 | 1 | 1 |
|  |  | 50 | 40 | 5 | 5 |
|  |  | 100 | 80 | 10 | 10 |
|  |  | 200 | 160 | 20 | 20 |
|  |  | 400 | 320 | 40 | 40 |
|  |  | 800 | 640 | 80 | 80 |
|  |  | 1000 | 800 | 100 | 100 |
|  |  | 2000 | 1600 | 200 | 200 |
|  |  | 3000 | 2400 | 300 | 300 |
| NCM622 | 10/6/2/2 | 0.1 | 0.06 | 0.02 | 0.02 |
|  |  | 1 | 0.6 | 0.2 | 0.2 |
|  |  | 10 | 6 | 2 | 2 |
|  |  | 50 | 30 | 10 | 10 |
|  |  | 100 | 60 | 20 | 20 |
|  |  | 200 | 120 | 40 | 40 |
|  |  | 400 | 240 | 80 | 80 |
|  |  | 800 | 480 | 160 | 160 |
|  |  | 1000 | 600 | 200 | 200 |
|  |  | 2000 | 1200 | 400 | 400 |
|  |  | 3000 | 1800 | 600 | 600 |
| NCM523 | 10/5/2/3 | 0.1 | 0.05 | 0.02 | 0.03 |
|  |  | 1 | 0.5 | 0.2 | 0.3 |
|  |  | 10 | 5 | 2 | 3 |
|  |  | 50 | 25 | 10 | 15 |
|  |  | 100 | 50 | 20 | 30 |
|  |  | 200 | 100 | 40 | 60 |
|  |  | 400 | 200 | 80 | 120 |
|  |  | 800 | 400 | 160 | 240 |
|  |  | 1000 | 500 | 200 | 300 |
|  |  | 2000 | 1000 | 400 | 600 |
|  |  | 3000 | 1500 | 600 | 900 |
| NCM111 | 10/3.3/3.3/3.3 | 0.1 | 0.033 | 0.033 | 0.033 |
|  |  | 1 | 0.33 | 0.33 | 0.33 |
|  |  | 10 | 3.33 | 3.33 | 3.33 |
|  |  | 50 | 16.67 | 16.67 | 16.67 |
|  |  | 100 | 33.33 | 33.33 | 33.33 |
|  |  | 200 | 66.67 | 66.67 | 66.67 |
|  |  | 400 | 133.33 | 133.33 | 133.33 |
|  |  | 800 | 266.67 | 266.67 | 266.67 |
|  |  | 1000 | 333.33 | 333.33 | 333.33 |
|  |  | 2000 | 666.67 | 666.67 | 666.67 |

**Table S13.** Experimental design for the mixture system of Li, Ni, Co, and Mn at *IC50* concentration with reduced proportion of Ni, or Co, or Mn.

| **Group** | **Proportion of Li/Ni/Co/Mn** | **LiCl (μM)** | **NiCl_2_·6H_2_O (μM)** | **CoCl_2_·6H_2_O (μM)** | **MnCl_2_·4H_2_O (μM)** |
| --- | --- | --- | --- | --- | --- |
| Control | 0/0/0/0 | 0 | 0 | 0 | 0 |
| 1 | 10/8/1/1 | 957.2196 | 765.7757 | 95.7220 | 95.7220 |
|  | 10/7/1/1 | 957.2196 | 670.0537 | 95.7220 | 95.7220 |
|  | 10/8/0/1 | 957.2196 | 765.7757 | 0 | 95.7220 |
|  | 10/8/1/0 | 957.2196 | 765.7757 | 95.7220 | 0 |
| 2 | 10/5/2/3 | 887.2415 | 443.6207 | 177.4483 | 266.1724 |
|  | 10/4/2/3 | 887.2415 | 354.8966 | 177.4483 | 266.1724 |
|  | 10/5/1/3 | 887.2415 | 443.6207 | 88.7241 | 266.1724 |
|  | 10/5/2/2 | 887.2415 | 443.6207 | 177.4483 | 177.4483 |
| 3 | 10/6/2/2 | 543.1419 | 325.8852 | 108.6284 | 108.6284 |
|  | 10/5/2/2 | 543.1419 | 271.5710 | 108.6284 | 108.6284 |
|  | 10/6/1/2 | 543.1419 | 325.8852 | 54.3142 | 108.6284 |
|  | 10/6/2/1 | 543.1419 | 325.8852 | 108.6284 | 54.3142 |
| 4 | 10/3.3/3.3/3.3 | 893.7998 | 297.9333 | 297.9333 | 297.9333 |
|  | 10/2.3/3.3/3.3 | 893.7998 | 208.5533 | 297.9333 | 297.9333 |
|  | 10/3.3/2.3/3.3 | 893.7998 | 297.9333 | 208.5533 | 297.9333 |
|  | 10/3.3/3.3/2.3 | 893.7998 | 297.9333 | 297.9333 | 208.5533 |

**Table S14.** Concentration-response curves parameters of Lorentz.

| **Indicator** | **Element** | **IRmax** | **A** | **w** | **EC_50_** |
| --- | --- | --- | --- | --- | --- |
| Inhibition on cell viability | Li | 2.81152 | -3256.78409 | 1320.6245 | 564.50974 |

Lorentz equation: IR= IRmax + (2*A/π)*(w/(4*(c-EC_50_)^2 + w^2))

**Table S15.** Concentration-response curves parameters of Logistic.

| **Indicator** | **Element** | **IRmin** | **IRmax** | **p** | **EC_50_** |
| --- | --- | --- | --- | --- | --- |
| Inhibition on cell viability | Ni | 2.7448 | 98.7849 | 6.7782 | 1230.7437 |
| Inhibition on cell viability | Co | 1.3062 | 98.4456 | 2.9037 | 275.5843 |
| Inhibition on cell viability | Mn | 5.8002 | 66.3628 | 3.2729 | 1245.4725 |
| Inhibition on cell viability | Li_10_Ni_8_Co_1_Mn_1_ | 2.0266 | 111.4549 | 2.5032 | 1463.3665 |
| Inhibition on cell viability | Li_10_Ni_5_Co_2_Mn_3_ | 2.7755 | 98.7173 | 3.3658 | 1182.6497 |
| Inhibition on cell viability | Li_10_Ni_6_Co_2_Mn_2_ | 5.1392 | 93.6392 | 5.6308 | 1147.0221 |
| Inhibition on cell viability | Li_10_Ni_3.3_Co_3.3_Mn_3.3_ | 2.1471 | 94.2704 | 8.6396 | 1110.6971 |
| Inhibition on mitochondrial metabolic activity | Li | -14.4822 | -31.7456 | 1.3109 | 259.8620 |
| Inhibition on mitochondrial metabolic activity | Ni | -0.1341 | 85.5001 | 1.0898 | 271.2357 |
| Inhibition on mitochondrial metabolic activity | Co | -23.8700 | 78.3894 | 2.1870 | 207.7813 |
| Inhibition on mitochondrial metabolic activity | Mn | -12.6202 | 97.0164 | 0.9221 | 1340.0641 |
| Inhibition on mitochondrial metabolic activity | Li_10_Ni_8_Co_1_Mn_1_ | 4.4518 | 122.9492 | 1.3526 | 1355.9204 |
| Inhibition on mitochondrial metabolic activity | Li_10_Ni_3.3_Co_3.3_Mn_3.3_ | 1.1490 | 100.0577 | 1.9789 | 904.8890 |

Logistic equation: IR = IRmax + (IRmin- IRmax)/(1 + (c/EC_50_)^p)

**Table S16.** Concentration-response curves parameters of Logistic5.

| **Indicator** | **Element** | **IRmin** | **IRmax** | **p** | **s** | **EC_50_** |
| --- | --- | --- | --- | --- | --- | --- |
| Inhibition on mitochondrial metabolic activity | Li_10_Ni_5_Co_2_Mn_3_ | -8.4849 | 87.9462 | 14.4227 | 0.0662 | 1498.0481 |
| Inhibition on mitochondrial metabolic activity | Li_10_Ni_6_Co_2_Mn_2_ | 6.8677 | 91.5631 | 29.5291 | 0.0177 | 1981.7425 |

Logistic5 equation: IR = IRmin + (IRmax - IRmin) / (1 + (EC_50_/c)^h )^s

**Table S17.** IAI model parameters.

| **Element mixture** | **K_Ni_** | **K_Co_** | **K_Mn_** | **IC_50,Ni_** | **IC_50,Co_** | **IC_50,Mn_** | ***p'*** |
| --- | --- | --- | --- | --- | --- | --- | --- |
| Li_10_Ni_8_Co_1_Mn_1_ | 0.3889 | 0.3361 | 1.9103 | 372.3090 | 321.7393 | 1828.5483 | 1.3996 |
| Li_10_Ni_5_Co_2_Mn_3_ | 0.4196 | 0.3626 | 2.0609 | 372.3090 | 321.7393 | 1828.5483 | 1.3996 |
| Li_10_Ni_6_Co_2_Mn_2_ | 0.6855 | 0.5924 | 3.3666 | 372.3090 | 321.7393 | 1828.5483 | 1.3996 |
| Li_10_Ni_3.3_Co_3.3_Mn_3.3_ | 0.4165 | 0.3600 | 2.0458 | 372.3090 | 321.7393 | 1828.5483 | 1.3996 |

$$IR\left( c_{\mathrm{Li}10\mathrm{Ni}8\mathrm{Co}1\mathrm{Mn}1} \right)=\frac{100}{1+\frac{1}{\left( \frac{0.3889\times0.8{\times c}_{\mathrm{Li}10\mathrm{Ni}8\mathrm{Co}1\mathrm{Mn}1}}{372.3090}+\frac{0.3361\times0.1{\times c}_{\mathrm{Li}10\mathrm{Ni}8\mathrm{Co}1\mathrm{Mn}1}}{321.7393}+\frac{1.9103\times0.1{\times c}_{\mathrm{Li}10\mathrm{Ni}8\mathrm{Co}1\mathrm{Mn}1}}{1828.5483} \right)^{1.3996}}}$$

$$IR\left( c_{Li10Ni5Co2Mn3} \right)=\frac{100}{1+\frac{1}{\left( \frac{0.4196\times0.5{\times c}_{Li10Ni5Co2Mn3}}{372.3090}+\frac{0.3626\times0.2{\times c}_{Li10Ni5Co2Mn3}}{321.7393}+\frac{2.0609\times0.3{\times c}_{Li10Ni5Co2Mn3}}{1828.5483} \right)^{1.3996}}}$$

$$IR\left( c_{Li10Ni6Co2Mn2} \right)=\frac{100}{1+\frac{1}{\left( \frac{0.6855\times0.6{\times c}_{Li10Ni6Co2Mn2}}{372.3090}+\frac{0.5924\times0.2{\times c}_{Li10Ni6Co2Mn2}}{321.7393}+\frac{3.3666\times0.2{\times c}_{Li10Ni6Co2Mn2}}{1828.5483} \right)^{1.3996}}}$$

$$IR\left( c_{Li10Ni3.3Co3.3Mn3.3} \right)=\frac{100}{1+\frac{1}{\left( \frac{0.4165 \times0.33{\times c}_{Li10Ni3.3Co3.3Mn3.3}}{372.3090}+\frac{0.3600 \times0.33{\times c}_{Li10Ni3.3Co3.3Mn3.3}}{321.7393}+\frac{2.0458 \times0.33{\times c}_{Li10Ni3.3Co3.3Mn3.3}}{1828.5483} \right)^{1.3996}}}$$

**Table S18.** Demographic and characteristics of population cohort (*n* = 31).

| **Variable** |  |
| --- | --- |
| Age (year, mean ± SD) | 42.06 (7.76) |
| Sex (Male, %) | 80.65 |
| Working years (year, mean ± SD) | 3.39 (2.07) |

**Table S19.** Concentrations of Li, Ni, Co, and Mn (μM) in whole blood.

| **Number** | **C_Li_** | **C_Ni_** | **C_Co_** | **C_Mn_** |
| --- | --- | --- | --- | --- |
| 1 | 1.8137 | 0.1257 | 0.0564 | 0.0766 |
| 2 | 0.1794 | ND | 0.0087 | 0.1283 |
| 3 | 0.0944 | 0.0704 | 0.0094 | 0.1846 |
| 4 | 1.6036 | 0.6519 | 0.0208 | 0.2288 |
| 5 | 1.9424 | 0.4128 | 0.0315 | 0.2383 |
| 6 | 1.8452 | ND | 0.0127 | 0.2517 |
| 7 | 1.1183 | 1.3332 | 0.0409 | 0.2520 |
| 8 | 1.5899 | 0.3058 | 0.0123 | 0.2564 |
| 9 | 2.2964 | 0.1718 | 0.0302 | 0.2591 |
| 10 | 0.3780 | ND | 0.0267 | 0.2921 |
| 11 | 0.6765 | ND | 0.0179 | 0.2942 |
| 12 | 0.5092 | 0.1104 | 0.1326 | 0.3220 |
| 13 | 1.1040 | ND | 0.0189 | 0.3237 |
| 14 | 1.0451 | 0.2555 | 0.0149 | 0.3471 |
| 15 | 0.7405 | 0.2607 | 0.0057 | 0.3699 |
| 16 | 0.6326 | 0.6339 | 0.0196 | 0.3724 |
| 17 | 0.6244 | ND | 0.0766 | 0.4383 |
| 18 | 0.7275 | 200.5956 | 0.0433 | 0.4542 |
| 19 | 0.4925 | 0.6722 | 0.0063 | 0.4623 |
| 20 | 0.5697 | 0.4211 | 0.0044 | 0.4660 |
| 21 | 1.5307 | 3.9720 | 0.0131 | 0.4748 |
| 22 | 1.1210 | ND | 0.0117 | 0.4838 |
| 23 | 1.0552 | 0.0222 | 0.0208 | 0.4928 |
| 24 | 1.2545 | 0.3586 | 0.0674 | 0.5037 |
| 25 | 0.7712 | 29.6431 | 0.0304 | 0.5588 |
| 26 | 0.6027 | 0.5128 | 0.0484 | 0.5698 |
| 27 | 1.0107 | 0.2371 | 0.0201 | 0.5966 |
| 28 | 0.2354 | 0.3260 | 0.0362 | 0.9169 |
| 29 | 0.5471 | ND | 0.0479 | 1.1790 |
| 30 | 0.5706 | 1.8431 | 0.1578 | 1.2040 |
| 31 | 0.7294 | 0.0860 | 0.0107 | 1.3062 |

ND, not detected.

**Reference**

1. L. Xia, C. Zhang, N. Lv, Z. Liang, T. Ma, H. Cheng, Y. Xia, L. Shi, Admsc-derived exosomes alleviate acute lung injury via transferring mitochondrial component to improve homeostasis of alveolar macrophages. *Theranostics* **12**, 2928-2947 (2022).
